# The cell colony development is connected with the accumulation of embryogenesis-related proteins and dynamic distribution of cell wall components in *in vitro* cultures of *Fagopyrum tataricum* and *Fagopyrum esculentum*

**DOI:** 10.1186/s12870-025-06119-3

**Published:** 2025-01-24

**Authors:** Magdalena Zaranek, Artur Pinski, Bozena Skupien-Rabian, Urszula Jankowska, Kamila Godel-Jedrychowska, Katarzyna Sala-Cholewa, Katarzyna Nowak, Ewa Kurczyńska, Ewa Grzebelus, Alexander Betekhtin

**Affiliations:** 1 Institute of Biology, Biotechnology and Environmental Protection, Faculty of Natural Sciences, University of Silesia in Katowice, St. Jagiellonska 28, Katowice, 40-032 Poland; 2 Proteomics and Mass Spectrometry Core Facility, Malopolska Centre of Biotechnology, Jagiellonian University, St. Gronostajowa 7A, Krakow, 30-387 Poland; 3 Department of Plant Biology and Biotechnology, Faculty of Biotechnology and Horticulture, University of Agriculture in Krakow, Ave. Mickiewicza 21, Krakow, 31-120 Poland

**Keywords:** Differentiation, *Fagopyrum*, Oleosins, Protoplast, Somatic embryogenesis, Transcription factors, Vicilins

## Abstract

**Background:**

Due to the totipotency of plant cells, which allows them to reprogram from a differentiated to a dedifferentiated state, plants exhibit a remarkable regenerative capacity, including under in vitro culture conditions. When exposed to plant hormones, primarily auxins and cytokinins, explant cells cultured in vitro can undergo differentiation through callus formation. Protoplast culture serves as a valuable research model for studying these processes in detail. This knowledge is particularly relevant for improving common and Tartary buckwheat species. To gain deeper insights into the stages of cell development from protoplasts—such as cell division, cell colony formation, and microcalli development—we focused on analyzing proteomes, cell wall composition, and changes in the expression profiles of selected genes in *Fagopyrum* protoplast cultures.

**Results:**

The results demonstrate a significant accumulation of somatic embryogenesis-related proteins like late embryogenesis abundant proteins (embryogenic protein-DC-8-like, seed biotin-containing protein) and endochitinases during the developmental path of protoplast-derived cultures. Additionally, we noted an extensive increase in seed storage proteins like vicilin, oleosins, and seed biotin-containing proteins during the culture. Investigation of somatic embryogenesis-associated transcription factors revealed massive up-regulation of *LEAFY COTYLEDON1* for the 50th day of *F. tataricum* protoplast-derived cultures. However, for *BABY BOOM*, the transcription factor was noted to be down-regulated during the development of cell colonies. Furthermore, we demonstrated the variable distribution of cell wall components like pectin side chains, arabinogalactan proteins (AGPs) and extensins (EXTs), indicating the reorganisation of cell wall composition during the culture period.

**Conclusions:**

This study revealed changes correlating with regaining embryogenic competence during the development of *Fagopyrum* protoplast-derived cell colonies. Our findings revealed variable expression levels of genes and proteins associated with somatic embryogenesis. This analysis identified an increase in seed storage proteins that play a significant role in the somatic somatic embryogenesis pathway of regeneration. Furthermore, the relationship between transcription factors and these processes seems to be connected with regaining somatic cells’ totipotency and promoting embryogenic competence of protoplast-derived cell colonies. Additionally, we observed dynamic changes in cell wall composition during the development of the protoplast-derived cultures.

**Clinical trial number:**

Not applicable.

**Supplementary Information:**

The online version contains supplementary material available at 10.1186/s12870-025-06119-3.

## Introduction

Buckwheat is an important under-used crop plant despite its noteworthy pharmaceutical and nutritional qualities. The most cultivated ones are common (*Fagopyrum esculentum*) and Tartary buckwheat (*Fagopyrum tataricum*), distinguished by the presence of phenolic compounds, minerals, dietary fibre and high-quality proteins with a well-balanced amino acid profile [1–3]. The main challenge in cultivating common buckwheat is their low and inconsistent yield, which results from self-incompatibility, short flower lifespan, embryo, seed, and flower abortion. The heterogeneity of maturation, seed shattering, preharvest sprouting, and sensitivity to biotic and abiotic stresses are the main cons of these two species, which are a challenge in buckwheat breeding programs [4]. An efficient Agrobacterium-mediated transformation and genome editing of *F. tataricum* was recently established, allowing for the improvement of agronomical traits [5]. We have also demonstrated protoplast-to-plant regeneration ability in cultures of common [6] and Tartary buckwheat [7]. This was achieved after supplementing a culture medium with phytosulfokine (PSK) and embedding protoplast in an agarose matrix. Additionally, the time needed for plant regeneration via protoplast cultures of buckwheat compared with other plants was faster, taking three to five months (common and Tartary buckwheat, respectively). Protoplast-to-plant regeneration opens the possibility of protoplast transfection and genetic modifications, especially by delivery of a ribonucleoprotein complex of Cas9-gRNA instead of a vector, resulting in transgene-free and homogenous plant [8], since regenerated plants originate from a single protoplast. However, researchers face challenges in achieving successful plant regeneration from single cells. The efficient process initially requires selecting the appropriate genotype and tissue to provide protoplasts capable of further development and regeneration. Choosing the proper protoplast culture conditions (liquid or semi-solid medium), hormones, and additional substances is crucial for overcoming the division latency of protoplasts. These factors also influence the culture’s embryogenic competence and regenerative potential [9].

Plant tissue plasticity enables cells to gain pluri- or totipotency, allowing their development into plants from single cells [10]. However, little is known about what happens and how differentiated cells reprogram and regenerate [11]. Protoplasts are an excellent example of gaining totipotency as differentiated cells undergo enzymatic cell wall removal and stress conditions, leading to cell dedifferentiation processes. Protoplasts are susceptible to surrounding conditions, such as hormones, that can create their development path. Enriched liquid medium for protoplast culture enables *de novo* cell wall synthesis and reinitiating of cell division [12]. Protoplast cell wall reconstruction is the main point of interest for many researchers [13–17]. The scaffold of cellulose microfibrils, cross-linked by hemicelluloses and embedded in the matrix of pectic polysaccharides, provides cell wall structure and physical properties [18]. Additionally, hydroxyproline-rich glycoproteins such as AGPs and EXTs are responsible for cell wall dynamics and mechanical properties [19]. Besides these, a cell wall can regulate morphogenetic processes and maintain differentiated cellular fate. Moreover, changes in the chemical composition of cell walls could be a marker of changes in the differentiation direction during the somatic embryogenesis process and plant growth [20]. Fagopyrum’s protoplast cell wall reconstruction pattern was recently shown [13].

Lipids are crucial in in vitro cultures, particularly in somatic embryogenesis. They are essential components of cell membranes, signalling molecules, and energy reserves. During somatic embryogenesis, lipids are involved in the formation and development of embryonic structures. The accumulation of lipid bodies often marks embryo development. Moreover, alterations in lipid metabolism and composition can influence the efficiency of embryogenesis, making lipids important regulators in the process. Studying lipid dynamics provides valuable insights into in vitro culture systems and can improve embryogenesis outcomes [21, 22].

However, far too little attention has been paid to the processes occurring during the middle and late stages of protoplast-derived cell colonies. Most studies focus on gene expression and proteome analysis of the first hours or the first week of culture. Characterisation of the changes in the transcript profile during the early steps of dedifferentiation and reentry into the cell division process was reported by Chupeau, et al. 2013 [23]. Gene expression in culture of mesophyll-derived protoplasts was shown by Xu, et al. 2021 [11]. Wang, et al. 2017 [24] provide information about proteomic analysis of developmental reprogramming in protoplast-derived cultures of moss *Physcomitrella patens*. de Jong, et al. 2007 [25] also showed a proteome study of the first days of *Medicago truncatula* protoplasts, focusing on molecular changes during protoplast proliferation.

Our recent investigations into the morphogenic and embryogenic callus of *F. tataricum* and *F. esculentum* revealed distinct differences in the development of protoplast-derived cultures [6, 7]. We observed variations in the time required for microcallus formation and plant regeneration. Specifically, protoplast cultures of *F. tataricum* took a longer period (50 days) for microcallus formation compared to *F. esculentum* (30 days). Additionally, our previous study confirmed that plant regeneration from protoplast-derived cultures in *F. tataricum* occurs through both organogenesis and somatic embryogenesis, while in *F. esculentum*, it occurs solely via somatic embryogenesis. Until now, information about the processes occurring in culture’s middle and late periods derived from protoplasts is scarce. Therefore, our goal was to describe and better understand what happens during the formation of cell colonies (day 5th, 15th and 30/50th ) on the level of proteomes, cell wall composition, and gene expression profile starting from protoplast cultures of *F. tataricum* and *F. esculentum*.

## Results

### Morphology of the callus used as a material source for protoplast isolation

**Fig. 1:**
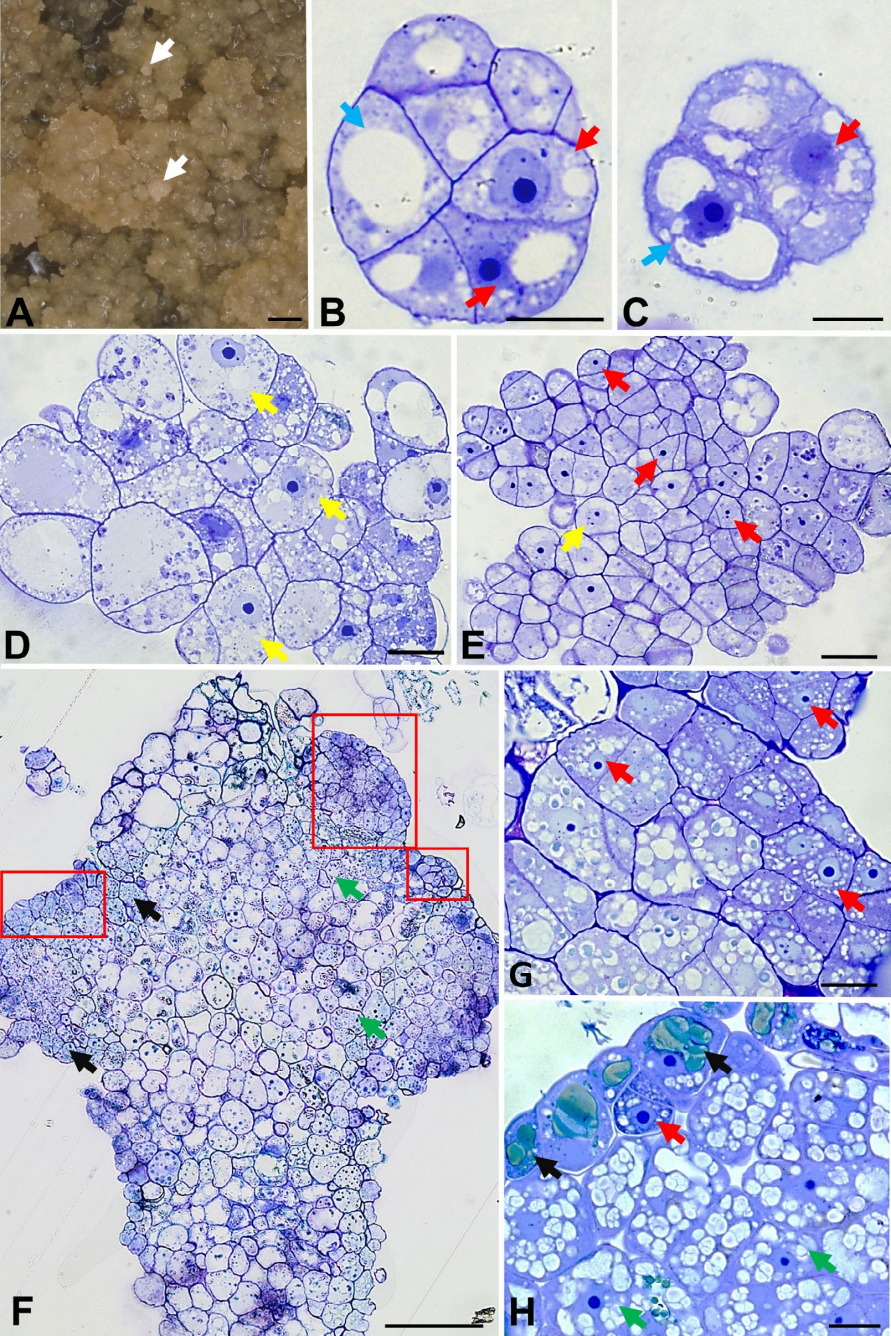
Morphology of one-week-old callus (**A**) and histological sections of the morphogenic callus-derived protoplast cultures of *F. tataricum* on the 5th (**B-C**), 15th (**D-E**) and 50th (**F-H**) day of the culture. White arrows (**A**) indicate pro-embryogenic cell complexes, blue arrows (**B**-**C**) point to vacuolated cells, red arrows (**B**,** C**,** E**,** G**,** H**) indicate meristematic cells; yellow arrows (**D**, **E**) show cells with multiple vacuoles and large nuclei; black arrows (**F**,** H**) indicate phenolic-containing cells; green arrows (**F**,** H**) demonstrate parenchymatous cells with small vacuoles and starch grains; red frame (**F**) marks regions rich in meristematic cells. Scale bars: 1 mm (**A**); 20 μm (**B-E**,** G-H**); 100 μm (**F**)

**Fig. 2:**
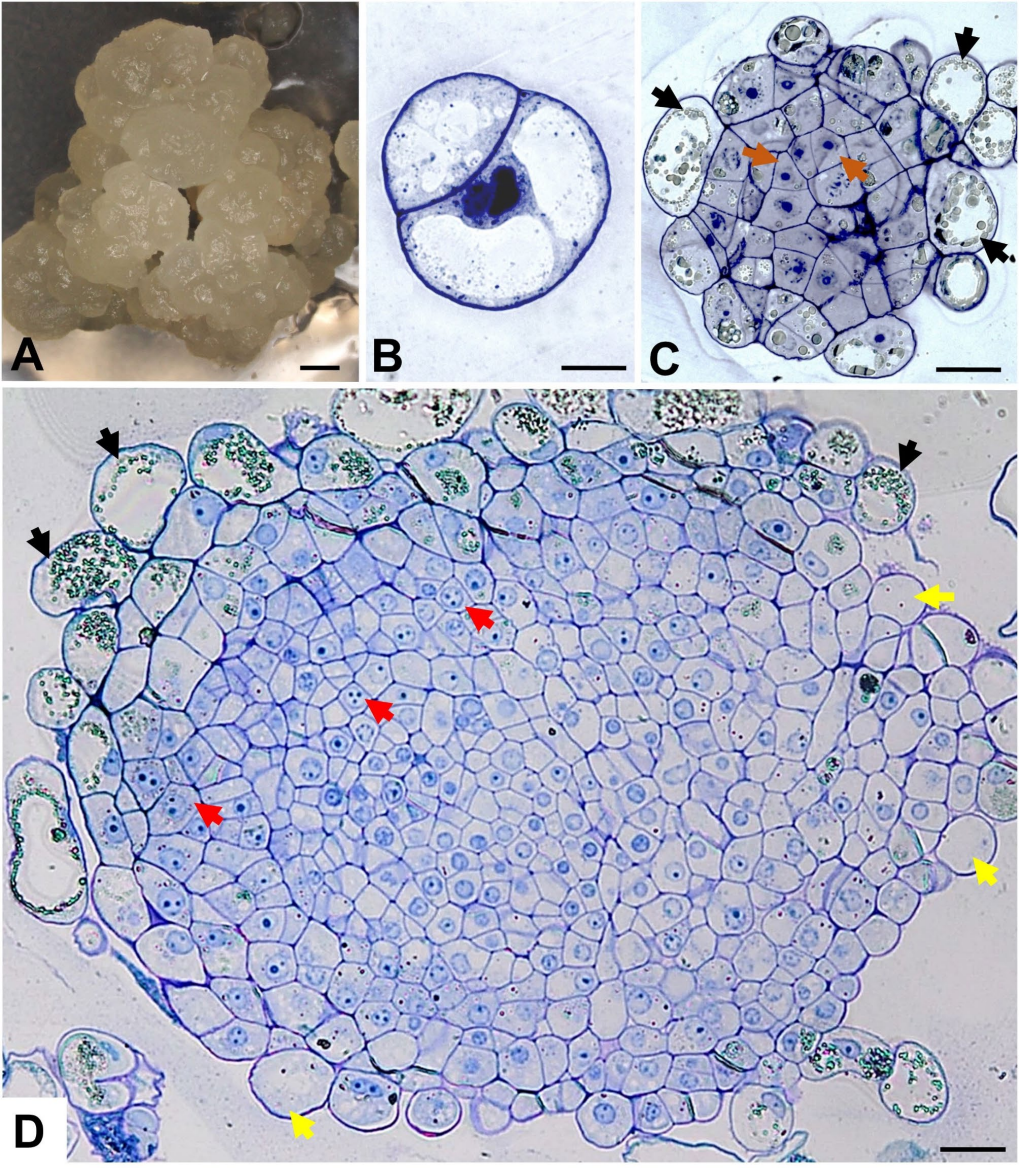
Morphology of two-week-old callus (**A**) and histological sections of the embryogenic callus-derived protoplast cultures of *F. esculentum* on the 5th (**B**), 15th (**C**) and 30th (**D**) day of the culture. Black arrows indicate phenolic-containing cells (**C-D**); orange arrows show embryogenic cells (**C**), red arrows point to meristematic cells (**D**); yellow arrows note the parenchymatous cells (**D**). Scale bars: 1 mm (**A**), 10 μm (**B**), 20 μm (**C-D**)

For protoplast cultures, the cells were isolated from the morphogenic callus (MC; Fig. 1A) of *F. tataricum* consisting of small pro-embryogenic cell complexes (PECCs; Fig. 1A white arrows) and ‘soft’ callus cells. In the embryogenic callus (EC) of *F. esculentum*, dense globular milky-white structures can be distinguished due to the accumulation of starch grains in the storage cells (Fig. 2A) [6, 7, 26]. The calli of both species differed in age: five-year-old for *F. tataricum* and two-year-old *F. esculentum* were used.

**Fig. 3:**
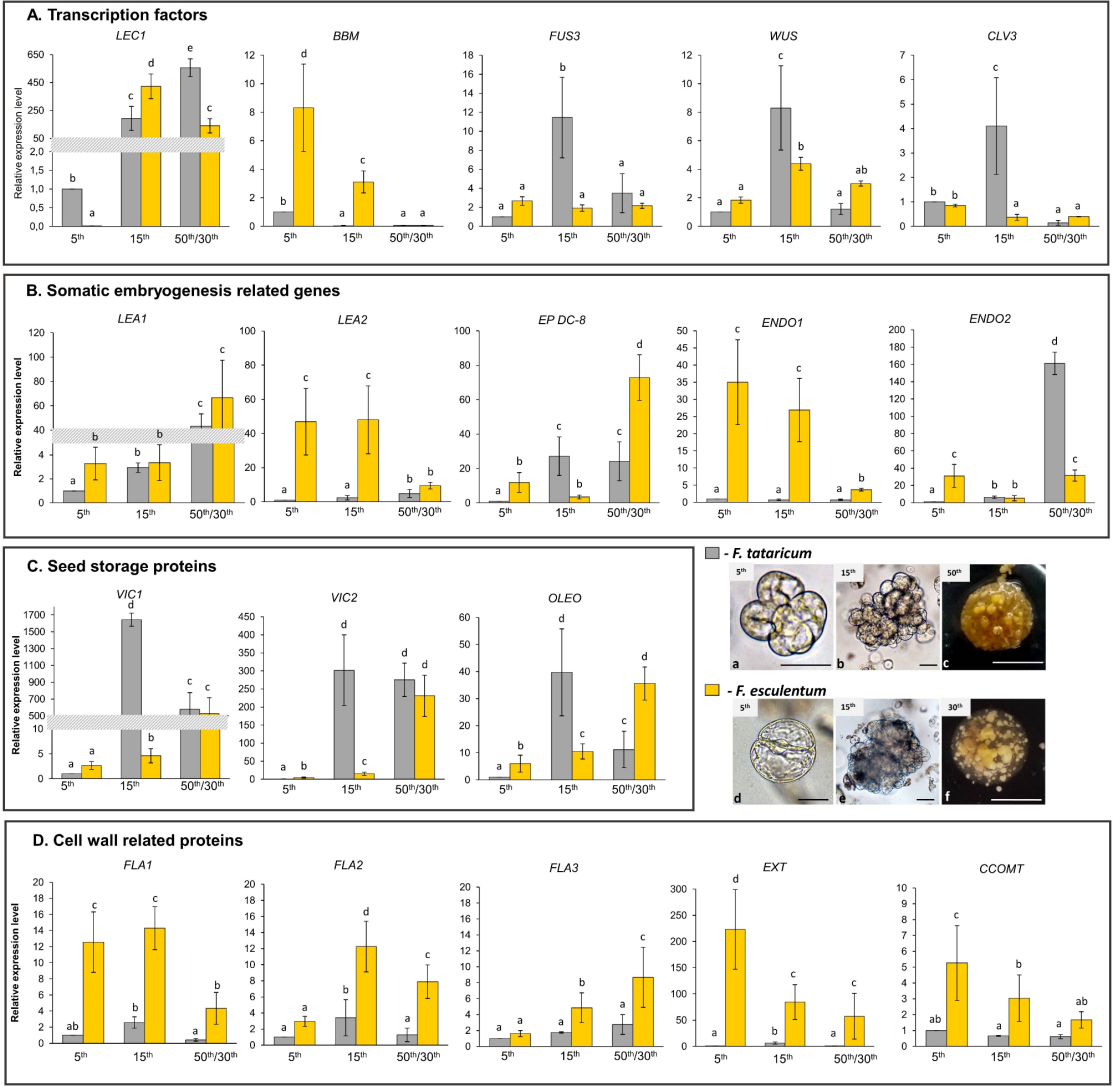
The expression level of (**A**) transcription factors, (**B**) somatic embryogenesis related genes, (**C**) genes coding seed storage proteins and (**D**) cell wall related proteins in the protoplast cultures of *F. tataricum* (day 5th, 15th, 50th ; gray bar charts) and *F. esculentum* (day 5th, 15th, 30th ; yellow bar charts). The expression level of genes was calibrated to expression at 5th culture of the *F. tataricum*. Different letters indicate a significant difference between time points and species according to Tukey’s HSD test (*p* < 0.05; *n* = 3; means ± SD are given). *LEAFY COTYLEDON* (*LEC1*); *BABY BOOM* (*BBM*); *FUSCA3* (*FUS3*); *WUSCHEL* (*WUS*); *CLAVATA3* (*CLV3*); *LATE EMBRYOGENESIS ABUNDANT PROTEIN* (*LEA*); *EMBRYOGENIC PROTEIN* DC-8-like (*EP DC-8*); *ENDOCHITYNASE* (*ENDO*); *VICILIN* (*VIC*); *OLEOSIN* (*OLEO*); *FASCICLIN-LIKE ARABONOGALACTAN PROTEIN* (*FLA*); *EXTENSIN* (*EXT*); *CAFFEOYL-COA O-METHYLTRANSFERASE* (*CCOMT*). The photo panel show the analysed time points of *F. tataricum* (**a**-**c**) and *F. esculentum* (**d**-**f**) protoplast cultures. Scale bars 50 μm (**a**-**b**, **d**-**e**), 5 mm (**c**, **f**)

**Fig. 4:**
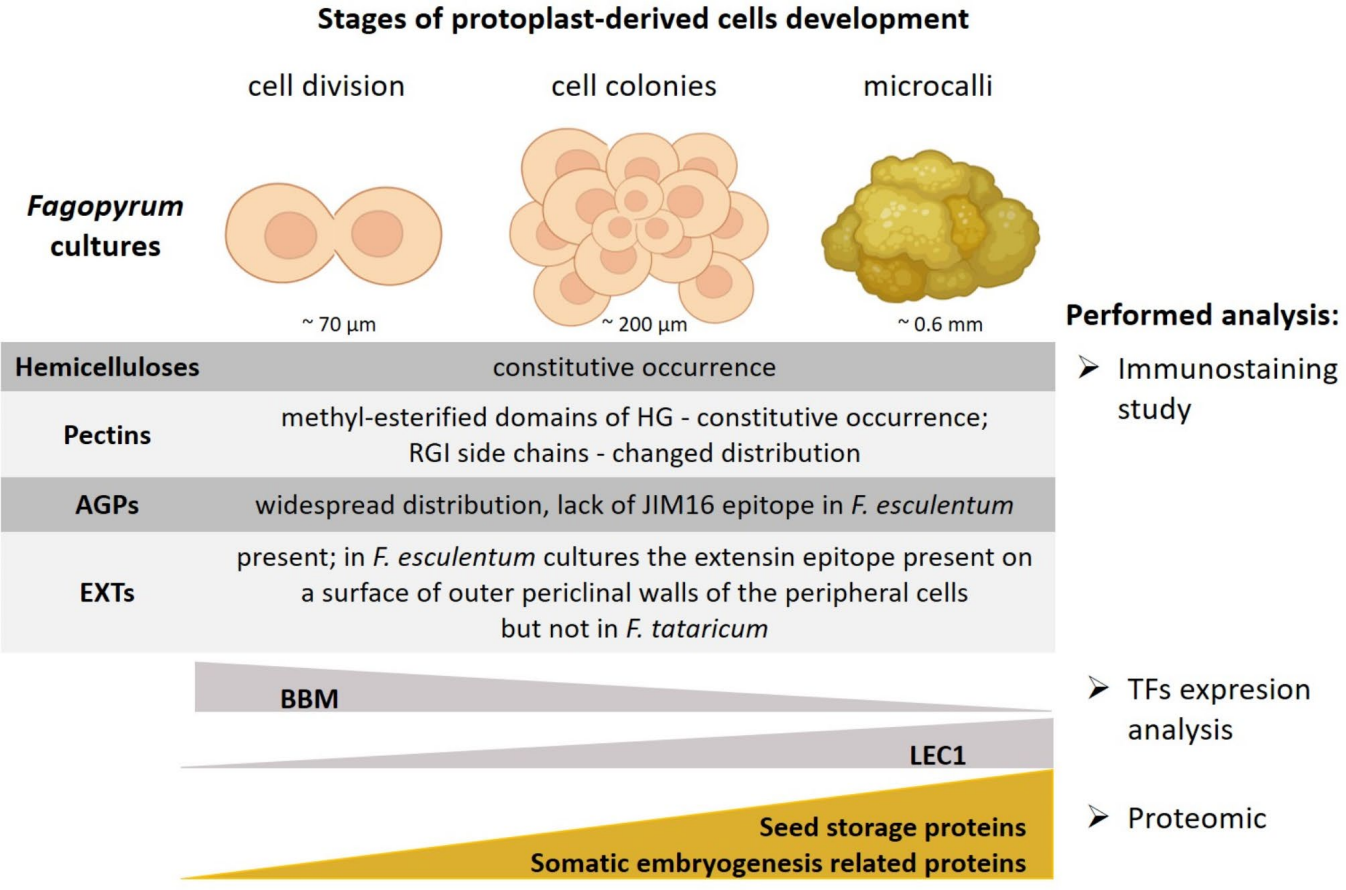
General overview on the analysed time points of the protoplast-derived cultures of *F. tataricum* and *F. esculentum*. The main results of the performed analysis are shown. Arabinogalactan protein (*AGPs*); *BABY BOOM* (*BBM*); extensins *(EXTs);* homogalacturonan *(HG); LEAFY COTYLEDON* (*LEC1*); rhamnogalacturonan I (*RGI*). This figure was created with http://BioRender.com. BioRender certificate confirming the publication rights is available upon request from the authors. The relative size of the stages of protoplast-derived cells is an approximation

For the analysis conducted in this study, three important time points of protoplast-derived cultures were selected. The events representing first cell division (5th day), cell colonies (15th day), and microcalli formation (30th for *F. esculentum* / 50th day for *F. tataricum*). The structures varied in size, measuring approximately 70 μm, 200 μm, and 0.6 mm, respectively (Figs. 3 and 4).

### Histological observations of protoplast-derived cell colonies

The histological observations of the culture at three time points (5th, 15th and 30/50th days) revealed differences in the size and number of cells that formed cell colonies (Fig. 1B-H). On the 5th day of *F. tataricum* culture, the structure consisted of parenchymatous-like cells with vacuoles of different sizes and numbers (Fig. 1B-C; blue arrows). However, some cell colonies cells exhibited certain meristematic cell features such as dense cytoplasm and a round-shaped, large nucleus with one or two nucleoli and smaller vacuoles (Fig. 1B, C; red arrows). On the 15th day, the cell colonies were composed mainly of thin-walled cells with numerous small vacuoles and large nuclei with one to three nucleoli (Fig. 1D-E; yellow arrows). Also, the occurrence of meristematic cells was noted (Fig. 1E; red arrows). Those cells were characterized by numerous small vacuoles and a large, round nucleus with one or more nucleoli. On the 50th day, the microcalli comprised various types of cells (Fig. 1F). Regions rich in meristematic cells were observed within the microcalli surface (Fig. 1F; red frames and Fig. 1G-H; red arrows) as well as phenolic-containing cells (PCCs) (Fig. 1F and H; black arrows). Parenchymatous cells with small vacuoles and starch grains (Fig. 1F and H; green arrows) and PCCs constituted microcallus central and most prominent part.

On the 5th day of *F. esculentum* culture, the cell colonies consisted mostly of cells after the first cell division (Fig. 2B). These cells had one or numerous vacuoles and sizeable nuclei. On the 15th and 30th day, the cell colonies and microcalli were characterized by different cells distributions than *F. tataricum* culture (Fig. 2C-D). On the 15th day, the structure consisted of embryogenic cells in the central part (Fig. 2C; orange arrows) and PCCs on the surface (Fig. 2C; black arrows), while *F. tataricum* culture did not contain PCCs at this time point (compare Fig. 1D-E, and Fig. 2C). On the surface of the 30th day microcall, PCCs with small phenolic droplets occurred (Fig. 2D; black arrows). The central part of microcalli was composed of meristematic cells, characterised by dense cytoplasm and round-shaped nuclei with one to three nucleoli (Fig. 2D; red arrows) and parenchymatous cells (Fig. 2D; yellow arrows).

### Immunocytochemical analysis

An immunocytochemical assay was performed to analyse the spatiotemporal distribution of selected cell wall components. Studied cell wall components included cellulose (visualised by FB28 staining), hemicellulose (xyloglucan, recognised by LM25 antibody; Supplementary Table S1), pectins (galactan, arabinan and homogalacturonan, recognized by LM5, LM6 and LM20 antibody, respectively; Supplementary Table S1) and hydroxyproline-rich glycoproteins such as AGPs (recognized by JIM13 and JIM16 antibody; Supplementary Table S1) and EXT (recognized by JIM20 antibody; Supplementary Table S1). The epitopes were selected based on previous work concerning rebuilding cell walls in protoplast cultures of these two buckwheat species [13]. Additionally, the localisation of selected AGPs and EXT was analysed in the MC of *F. tataricum*, which was used in our research as the donor material for protoplast isolation [26]. The results of epitopes distribution were summarised in Table 1 based on photographs presented in Supplementary Fig. S1-S14.

**Table 1:** Summary of the immunocytochemical detection of selected antigens in protoplast cultures of *F. tataricum* and *F. esculentum*

Antibody	Day of culture	Presence (+) or absence (-) of the analysed epitope		Signal localisation			
				Cell wall		Cytoplasmic compartments	
		*Ft*	*Fe*	*Ft*	*Fe*	*Ft*	*Fe*
**Hemicelluloses**							
LM25	5th	+	+	+	+	+	-
	15th	+	+	+	+	-	-
	50th /30th	+	+	+*	+	+	-
**Pectins**							
LM20	5th	+	+	+	+	-	-
	15th	+	+	+	+	-	-
	50th /30th	+	+	+*	+	-	-
LM5	5th	+	+	-	+	+	-
	15th	+	+	+	+	-	-
	50th /30th	+	+	+	+	-	-
LM6	5th	+	+	+*	+	+	+
	15th	+	+	+	+	-	-
	50th /30th	+	+	+	+	-	-
**Arabinogalactan proteins**							
JIM13	5th	+	+	+	+	+	+
	15th	+	+	+	+	+	+
	50th /30th	+	+	+	+	+	+
JIM16	5th	+	-	-	-	+	-
	15th	+	-	-	-	+	-
	50th /30th	+	-	-	-	+	-
**Extensin**							
JIM20	5th	+	+	+	+	-	-
	15th	+	+	+	-*	-	-
	50th /30th	+	+	+*	-*	-	-

*epitopes were noted on a surface of outer periclinal walls of the peripheral cells

*Ft - F. tataricum; Fe - F. esculentum*

Xyloglucan epitope was mainly distributed in the cell walls of *F. esculentum* and *F. tataricum* culture at each analysed time point (Supplementary Fig. S1, S2). The fluorescence signal was also present in cytoplasmic compartments (Supplementary Fig. S1A’-A”; red arrows) as well on the surface of outer periclinal walls of the peripheral cells (Supplementary Fig. S1C’-C”; brown arrow) in *F. tataricum*. Methyl-esterified homogalacturonan domains were localised in the cell wall at the analysed steps in both species cultured (Supplementary Fig. S3, S4). Additionally, the LM25 epitope was noted on the surface of the outer periclinal walls of the peripheral cells (Supplementary Fig. S3C’-C”; brown arrow) in *F. tataricum*.

LM5 epitope (β-1,4-galactan in side chains of rhamnogalacturonan I (RGI)) was detected differently (Supplementary Fig. S5, S6). On the 15th day, the LM5 epitope was distributed only in some inner cell walls of the *F. tataricum* cell colonies (Supplementary Fig. S5B-B”; yellow arrows) in contrast to *F. esculentum*, in which the epitope was localised mainly in the outer periclinal cell walls (Supplementary Fig. S6B’-B”, blue arrows). LM6 epitope (α-1,5-arabinan from side chains of RGI) occurred in cell walls (Supplementary Fig. S7, S8) as well as in cytoplasmic compartments (Supplementary Fig. S7A’-A” and S8A’-A”).

AGPs epitope detected by the JIM13 antibody was localised in the cell walls and cytoplasmic compartments at each of the analysed time points (Supplementary Fig. S9-S10). Nonspecific binding to phenolics, localised in phenolic-containing cells, was noted (Supplementary Fig. S9C’-C”, S10C’; purple arrows) as well as the presence of AGPs in the vacuole of *F. esculentum* culture occurred (Supplementary Fig. S10A’, B’; white arrows). The JIM16 epitope was mainly detected in the cytoplasmic compartments (Supplementary Fig. S11; red arrows) and vacuoles (Supplementary Fig. S11A’; white arrow) of *F. tataricum* in comparison to *F. esculentum* where the epitope was absent (Supplementary Fig. S12).

Extensin epitope, detected by JIM20 antibody, was present in the cell walls on the 5th day of *F. tataricum* (Supplementary Fig. S13A’-A”) and *F. esculentum* (Supplementary Fig. S14A’-A”) cultures in a dotted manner. As well, the distribution of the JIM20 epitope was restricted to the surface of the outer periclinal walls of the peripheral cells of *F. tataricum* (Supplementary Fig. S13C’; brown arrow) and *F. esculentum* (Supplementary Fig. S14B’ and C’; brown arrows).

### Lipid staining

The occurrence of lipid substances was demonstrated by Sudan Black and Sudan III staining, as both are used for staining triglycerides, lipids, and lipoproteins. However, Sudan Black can additionally stain phospholipids. A positive reaction is indicated by the orange colour for Sudan III and black or blue for Sudan Black [27].

In *F. tataricum* protoplast-derived cultures, the occurrence of lipids at each of the analysed time points was observed. Six hours after protoplast isolation, small lipid droplets were revealed in the cytoplasm compartments (Supplementary Fig. S15A-B; red and black arrows). On the 5th and 15th day of the culture, the lipid droplets had different sizes, either easily detectable (Supplementary Fig. S15C-F) or very small and dispersed throughout the cytoplasm (Supplementary Fig. S15C’-D’). On the 50th day of culture, the lipid droplets were often noted near the cell wall (Supplementary Fig. S16A, C). Staining by Sudan Black showed a higher amount of blue-colored lipid droplets (Supplementary Fig. S16D-E; red arrows) compared to the application of Sudan III, possibly due to the affinity of Sudan Black to phospholipid fraction.

In *F. esculentum* protoplast cultures, the staining of small lipid droplets was noted in protoplasts after isolation (Supplementary Fig. S17A-B). However, in some cells, bigger droplets were detected, and those were located near the plasma membrane (Supplementary Fig. S17B’). On the 5th day of culture, most of the cell colony were intensely coloured by Sudan Black (Supplementary Fig. S17C); however, in some, only a few lipid droplets were distributed near the cell wall (Supplementary Fig. S17C’). On the 15th day of culture, the lipids were present in the cytoplasm and the cell walls (Supplementary Fig. S17E; red arrows). The dark black color of the cell colonies indicated its dense cytoplasm. For Sudan III staining, only some orange droplets were noted in the cells of cell colonies (Supplementary Fig. S17F; black arrows). On the 30th day of culture, lipid storage was pointed out, especially in peripheral cells of the microcalli (Supplementary Fig. S18A-C), which was demonstrated by Sudan III staining. Sudan Black staining revealed the presence of more lipid droplets (Supplementary Fig. S18D-F) distributed throughout the cell’s cytoplasm and localised in cell walls (Supplementary Fig. S18D).

### Proteomics analysis

The proteomic analysis performed at three time points of protoplast-derived cultures allowed us to identify 3664 proteins in *F. esculentum* and 3811 proteins in *F. tataricum* (Supplementary Fig. S19A). In *F. tataricum*, on the 50th day of culture, we identified the highest number of proteins, 3046, followed by 2646 on the 5th day and 2344 on the 15th day. The 1622 proteins were shared between all timepoints in the culture of *F. tataricum*. Similarly, in *F. esculentum*, the highest number of proteins were identified on the 30th day of culture, with 2933 different proteins being identified, followed by 2947 proteins on the 5th day and 2321 proteins on the 15th day, with 1846 proteins shared between all time points. The comparative analysis revealed significantly over-accumulated and under-accumulated proteins between the days of culture in both species (Supplementary Fig. S19B), showing the most pronounces changes in the protein accumulation between the 5th and 30th /50th days of culture (for *F. tataricum*: 503 over-accumulated and 657 under-accumulated, for *F. esculentum*: 541 over-accumulated and 655 under-accumulated) and the smallest between the 15th and 30th /50th days of culture (for *F. tataricum*: 189 over-accumulated and 287 under-accumulated, for *F. esculentum*: 139 over-accumulated and 193 under-accumulated) (Supplementary Fig. S19B and C). In *F. esculentum*, the highest increase in protein accumulation was observed for the sucrose-cleaving enzyme (33.3-times in comparison of 15th vs. 5th, 188.7-times for 30th vs. 15th ) (Supplementary Table S2, sheet 1) and the most significant decrease for embryonic protein DC-8-like (EP DC-8) (22.6-times in comparison of 15th vs. 5th ) (Supplementary Table S2, sheet 2 and Table 2). In *F. tataricum*, the highest increase in protein accumulation was observed for seed biotin containing protein (SBP) (94.1 times in comparison of 50th vs. 5th, 163 times for 50th vs. 15th ) (Supplementary Table S2, sheet 1) and the most significant decrease for caffeoyl-CoA O-methyltransferase (CCOMT) (35.1-times in comparison of 15th vs. 5th ) (Supplementary Table S2, sheet 2 and Table 2). The Gene Ontology (GO) term enrichment of proteins present in both species at analysed timepoints showed 61 enriched terms shared by all analysed samples (12 molecular functions (MF), 22 cellular compartments (CC), and 27 biological processes (BF)), among them GO terms related to cell wall (CC, GO:0005618), plasmodesma (CC, GO:0009506), and response to cytokinin (BP, GO:0009735) (Supplementary Table S2, sheet 3 and 4). On the 5th day, two enriched GO terms were shared by both species: vesicle organisation (BP, GO:0016050) and TBP-class protein binding (MF, GO:0017025). On the 15th day, three enriched GO terms were shared by *F. esculentum* and *F. tataricum*: response to anoxia (BP, GO:0034059), intracellular protein-containing complex (CC, GO:0140535), transporter complex (CC, GO:1990351). For the 30th /50th day of culture, 12 GO terms were enriched, among others: response to desiccation (BP, GO:0009269), cellular oxidant detoxification (BP, GO:0098869), and peroxisome (CC, GO:0005777).

**Table 2:** Differentially accumulated proteins (DAPs) of *F. tataricum* and *F. esculentum* protoplast cultures

Majority protein IDs Ft/Fe	Name	Abbreviation	*F. tataricum*			*F. esculentum*		
			15th vs. 5th	50th vs. 5th	50th vs. 15th	15th vs. 5th	30th vs. 5th	30th vs. 15th
**Somatic embryogenesis related proteins**								
GWHPBJBL008574/ GWHPBJBK010836	Embryogenic protein DC-8-like	EP DC-8	NS	NS	28.5	-22.6	-1.8	12.6
GWHPBJBL018466/ GWHPBJBK015397	Endochitinase	ENDO1	NS	NS	NS	NS	NS	NS
GWHPBJBL018472/ GWHPBJBK015393		ENDO2	NS	NS	NS	NS	NS	NS
**Seed storage proteins**								
GWHPBJBL016675/ GWHPBJBK015259	Vicilin	VIC1	NS	136.1	100.8	NS	35.7	41.4
GWHPBJBL032170/ GWHPBJBK037628		VIC2	NS	84	101.6	NS	71.7	88.7
GWHPBJBL013999/ GWHPBJBK017428	Oleosin	OLEO	NS	24.3	6.0	NS	9.5	25.4
**Cell wall related proteins**								
GWHPBJBL002808/ GWHPBJBK004524	Fasciclin-like arabinogalactan protein	FLA1	NS	NS	NS	NS	NS	NS
GWHPBJBL002736/ GWHPBJBK004425		FLA2	NS	NS	NS	NS	3.9	4.7
GWHPBJBL013649/ GWHPBJBK018791		FLA3	NS	4.7	4.9	NS	NS	4.3
GWHPBJBL027662/ GWHPBJBK030169	Extensin	EXT	NS	NS	NS	-2.9	-2.3	NS
GWHPBJBL024749/ GWHPBJBK027741	Caffeoyl-CoA O-methyltransferase	CCOMT	-35.1	NS	NS	-4.1	NS	NS

*NS* – non significant

### Genes expression analysis

We analysed the expression level of genes related to plant regeneration: *LEAFY COTYLEDON1* (*LEC1*), *BABY BOOM* (*BBM*), *FUSCA 3* (*FUS3*), *WUSCHEL* (*WUS*), *CLAVATA* (*CLV3*) and genes encoding proteins that are accumulated at a high level during protoplast-derived cultures, such as *LATE EMBRYOGENESIS ABUNDANT PROTEIN* (*LEA1, LEA2*), *EP DC-8*, *ENDOCHITYNASES* (*ENDO1*,* ENDO2*), *VICILIN* (*VIC1*,* VIC2*), *OLEOSIN* (*OLEO*), *FASCICLIN-LIKE ARABINOGALACTAN PROTEINS* (*FLAs*), *EXTENSINE* (*EXT*), *CCOMT*. The expression of all analysed genes was calibrated to the 5th day of *F. tataricum* culture to reveal the difference in expression level between time points of the culture and species (Fig. 3). We observed the higher expression of *BBM*,* LEA2*,* ENDO1*,* CCOMT*,* FLA1*,* 2*,* 3*,* EXT* genes at almost all analysed time points of the *F. esculentum* culture compared *to F. tataricum*. The most intense upregulation of expression was observed for the *EXT* gene on the 5th day of *F. esculentum* culture, where the expression was over 200 times higher than at *F. tataricum* culture. In contrast, lower expression of *VIC1* and *VIC2* genes was observed during the *F. esculentum* culture, and transcription of both genes was strongly stimulated on the 15th day of the *F. tataricum* culture. The differences in expression of other analysed genes, such as *FUS3*,* WUS*,* CLV3*, *LEA1*,* EP DC-8*,* ENDO2*,* and OLEO*, between the species were indicated at time points depending on genes. In summary, the expression pattern and level of analysed genes differed between species and time points of the culture.

## Discussion

Plant cells devoid of cell walls are characterised by acquiring or regaining totipotency due to the possibility of reprogramming from a differentiated to a dedifferentiated state and high regeneration ability [28]. Last year, we confirmed the ability of common and Tartary buckwheat protoplasts for regeneration [6, 7]. Differences in the time required for microcalli formation and plant regeneration were noted. Therefore, we focused on determining whether differences, shared aspects, or potentially universal processes are involved. Research has focused chiefly on transcriptome and proteomic analysis of developmental reprogramming in protoplast-derived cultures [11, 23–25], while the fate of protoplast during development and gaining totipotency remained unexplored. Analysis of proteome, cell wall composition, and changes in the expression profile of the selected genes in *Fagopyrum* protoplast-derived cultures brought the missed information about plant cells’ lack of cell wall ability to develop and form higher structures like cell colonies, microcalli and plants.

### Transcription factors and proteins related to somatic embryogenesis

Among various factors that control plant cell reprogramming and are involved in gaining embryogenic competence, the major genes encoding transcription factors (TFs) like *LECs*,* BBM*,* WUS*,* CLV3*, and *FUS3* were studied [10, 29, 30]. The *LEC1* gene is essential for in vitro somatic embryogenesis induction and acquisition of embryogenic competence and controls many aspects of plant embryogenesis [31–33]. The present work provides a relation between *LEC1* expression and induction of somatic embryogenesis. Also, it explains events that occur during protoplast reprogramming at three time points (day 5th, 15th and 30th /50th ) of *Fagopyrum* protoplast-derived cultures.

*LEC1* is a sugar metabolism regulator by activating *SUCROSE SYNTHASE 2* (*SUS2*) [34]. The non-hormonal inducers of embryo development belong to sucrose concentration [35]; thus, SUS2 control somatic embryogenesis, which was proposed by Rolland, et al. 2002 [36]. Our proteomic results indicate a significant accumulation of SUCROSE SYNTHASE (SUS) on the 15th day of *F. esculentum* culture, which correlates with high expression of *LEC1* (Fig. 3A). According to Stein, et al. 2019 [37] and Ruan 2007 [38] *SUS* is required for the turgor build-up necessary for cell elongation, cellulose synthesis for cell walls and deposition of callose in the plasmodesmata. We can speculate that the identified enzyme can be significant for sugar metabolism in relation to the development of protoplast cultures that go through dynamic metabolic changes. Additionally, *LEC1* controls the expression of seed storage proteins (SSPs) genes by regulation of *ABSCISIC ACID INSENSITIVE 3* (*ABI3*) and *FUS3* genes [39]. Interestingly, we observed a greatly increased expression of VIC1 and VIC2 with cultivation time (Table 2), belonging to a storage protein that correlates with *LEC1* activity (compare Fig. 3A with C). In oil palm, vicilin-like proteins were upregulated in high-embryogenic ortets and were suggested as markers of embryogenic tissue. Sahara, et al. 2023 [40] proposed that low expression of VICs in the oil palm callus can result in low-embryogenic induction. Interestingly, we observed a high increase in the expression of *VIC1* and *VIC2* along with cultivation time (Fig. 3C). Therefore, we can suspect that increasing expression of *VIC* genes in the *Fagopyrum* cultures can be related to somatic embryo formation. Moreover, overexpression of *LEC1* with *ABI3* and *FUS3* leads to globally higher expression of fatty acid synthesis genes and significantly increased levels of fatty acid compounds in *Arabidopsis* [41]. We noted a high accumulation of OLEOSIN proteins (structural proteins of oil bodies) on the 15th day of *F. tataricum* and the 30th day of *F. escilentum* cultures (Table 2), which correlates with high expression of *LEC1* (compare Fig. 3A with C). Gliwicka, et al. 2012 [42] showed that higher expression of the *OLEOSIN4* (*OLEO4*) gene in embryogenic cultures of *Arabidopsis* compared to non-embryogenic ones and inactivation of *OLEO4* gene resulted in impaired somatic embryogenesis induction. The researchers suggested that *OLEO4* genes may support the tissue’s perception of embryogenic signals, leading to embryogenic development. Dutta, et al. 1991 [22] revealed a higher proportion of lipid body area in somatic embryos than in callus and suspension culture cells. The high presence of OLEO proteins in *Fagopyrum* protoplast cultures can indicate an increase in oil bodies, thus leading to preparation for somatic embryogenesis events. Additionally, Potocka, et al. 2012 [43] showed that staining *Arabidopsis* explants with lipophilic dyes revealed lipid lamellae within some cell walls, which correlate with lipid transfer protein 1 distribution, pointing to morphogenic events occurring during somatic embryogenesis. In our investigation, lipids staining did not show any significant differences between the analysed time points, and we noted abundant phospholipids occurrence in *Fagopyrum* protoplast cultures.

The *LEC1* activity can also be connected with processes occurring during protoplast cultures, like cell elongation. Junker, et al. 2012 [44] demonstrated that *LEC1* targets cell-wall modification- and hormone- related genes leading to hypocotyls elongation of *Arabidopsis*. It was revealed that *LEC1* targets genes coding enzymes that modify cell walls, like xyloglucan hydrolase and expansins, which are involved in cell wall modification; thus, cell expansion processes can be observed during the development of protoplast-derived cultures.

Another TF belonging to the LECs group of genes is FUS3, which plays a key role in seed development, somatic embryos and plant lateral organ formation [45]. Moreover, *FUS3* can enhance the competence for somatic embryogenesis by decreasing the level of gibberellin, ethylene, and activation of *YUC* genes responsible for auxin biosynthesis [31]. *FUS3* is also a key regulator in accumulating SSPs and other reserve materials like lipids and carbohydrates [45]. We observed significantly increased expression of *FUS3* on the 15th day of *F. tataricum* culture, which correlated with the highest accumulation of *OLEO* and *VICs* (compare Fig. 3A with C).

*BBM* carries important functions in proliferation and re-differentiation during embryogenesis, participating in regulating genes involved in controlling cell signalling, cell wall biosynthesis and modification [31, 46, 47]. Kulinska-Lukaszek, et al. 2012 [46] noted that the GUS gene expression under the BBM promoter in immature zygotic embryos of *Arabidopsis* was restricted to dividing cells and cell clusters. Authors postulated that *BBM* promotes cell proliferation and defines an undifferentiated state [48]. In our study, we observed the highest level of *BBM* expression among examined time points on the 5th day for *F. tataricum*, the 5th and 15th day of *F. esculentum* (Fig. 3A), which are significant time points for the first cell division events in *Fagopyrum* protoplasts-derived cultures [6, 7]. Thus, we can speculate that *BBM* activity was related to cell proliferation, cells re-entering the cell cycle and dividing cells. Moreover, the abovementioned processes relate to changes in the structure of the cell wall that can be regulated through genes and proteins targeted by *BBM*, such as actin DEPOLYMERIZING FACTOR 9, AGPs, and proteins participating in synthesis or modification of cell wall polysaccharides as shown for *Brassica napus* [48]. Interestingly, after the activation of the *BBM* gene in *Arabidopsis* mesophyll cells, the actin cytoskeleton’s reorganisation was observed, forming dense actin networks [48]. Such changes are characteristic of cells reorganising their cytoplasm before division (which occurs in protoplasts) or elongation. Among other genes regulated by *BBM*, Horstman, et al. 2017 [49] noted *LECs* genes, which show a relation between the *BBM*- and *LEC*-mediated somatic embryogenesis pathways. Therefore, we presume that the high activity of *BBM* on the 5th day of the cultures was associated with *BBM*-positive somatic embryogenesis regulations that next are taken over by *LEC1* (compare Fig. 3A). Moreover, *LEC1* and *BBM* are essential genes for embryo differentiation and maturation [47]. Together, this relation can suggests that *BBM* activity in *Fagopyrum* protoplast cultures may be related to different developmental pathways that occur close to the time of the first cell division and influence further embryo formation.

*WUS* and *CLV3* are meristem central regulators playing a pivotal role in determining stem cell fate in shoot apical meristem based on antagonistic function [50]. Furthermore, WUS prominent role is to maintain totipotent, embryogenic cell potential and to prevent their differentiation. Importantly, *WUS* expression is relevant for determining cell ability to become pluripotent or totipotent [30, 51]. Deyhle, et al. 2007 [52] observed that *WUS* regulates cell differentiation due to early expression during anther development and terminated expression during cell differentiation. Results of Zuo, et al. 2002 [53] show that *WUS* is a key factor in maintaining or inducing embryogenic potential compared to *LEC1*, which is a driving force for embryo cell differentiation. In the *Fagopyrum* cultures, the highest level of *WUS* on the 15th day (Fig. 3A) was observed, which may relate to cells’ totipotent character after the second step of dedifferentiation (cell division). For many species, increased expression of *WUS* increases the ability to form somatic embryos and regeneration capacity [30, 47]. On the other hand, *WUS* activates the expression of *CLV3*, which inhibits its expression, causing the differentiation of meristematic cells [51]. In our results, we observed a correlation in the level of *WUS* and *CLV3* (significantly high level for *WUS* and *CLV* expression) on the 15th day of *F. tataricum* culture (compare Fig. 3A), which can indicate the feedback loop between these genes. According to Elhiti 2010 [54], genes repressing meristematic cell characters like CLV3 strongly reduce somatic embryogenesis; therefore, the low level of *CLV3* in *Fagopyrum* cultures correlates with the high regeneration ability.

### Somatic embryogenesis-related proteins

Among the most common proteins reported as potential markers of somatic embryogenesis [55] in *Fagopyrum* protoplast cultures, we distinguished the late embryogenesis abundant (LEA) proteins group. LEA proteins generally accumulate in response to biotic and abiotic stresses and provide a protective function. Conditions of *in vitro *culture and reactive oxygen species generate significant oxidative stress effects during the whole developmental period of protoplast cultures. These factors can correlate with increased levels of LEAs proteins like EP DC-8 and SBP (also belong to LEA group), which are highly accumulated in *Fagopyrum* cultures. We can suspect that LEA and SSPs can play a protective function and increase tolerance to stress factors in protoplast cultures. The dicot LEA protein DC8-like is known to be expressed in somatic or zygotic embryos but not in mature tissue; thus, according to Hatzopoulos, et al. 1990 [56], it is an embryo-specific gene. Additionally, many reports have shown that the accumulation of LEA and storage proteins is associated with embryogenic transition, leading to the induction of somatic embryogenesis [57]. Likely, the occurrence of LEAs, SSP, SBP and VIC in *Fagopyrum* cultures can coincide with events leading to somatic embryogenesis.

Endochitinases may influence somatic embryo development by releasing signal molecules [55]. In *Fagopyrum* protoplast cultures, we noted significantly increased expression of the endochitinases gene on the 50th day for *F. tataricum* (Fig. 3B). Hengel 1998 [58] demonstrated that endochitinases and AGPs can promote the formation of somatic embryos in protoplast cultures of carrot, which is related to the presence of endochitinases cleavage sites in AGPs, based on chitinase-like protein secretion in embryogenic and non-embryogenic cultures of *Dactylis glomerate* L. Tchorbadjieva, et al. 2006 [59] distinguished these proteins as marker of embryogenic potential. In accordance with this, we can suggest that signal molecules generated by endochitinases may affected somatic embryos formation in *Fagopyrum* cultures.

### Changes in the cell wall

Removal of the cell wall, which is the first step of protoplast isolation, leads to cell dedifferentiation; after that, wall re-establishment is the most prominent step for protoplasts to divide and develop into callus. The wall components such as cellulose, hemicellulose, and pectin are crucial for establishing and maintaining the cellular differentiation status by maintaining cell-cell communication [12, 20]. As it was postulated by Majewska-Sawka, et al. 2003 [15] and Wiśniewska, et al. 2008 [16], cell wall composition is one of the important factors controlling plant regeneration *via* the protoplast cultures. For *Fagopyrum* protoplasts-derived cultures, Sala-Cholewa, et al. 2024 [13] described the mechanism of cell wall re-synthesis and pointed out differences in the spatio-temporal appearance or disappearance of individual epitopes during the first 72 h of protoplast cultures. Therefore, our goal was to investigate the cell wall composition and distribution of their components in the reconstituted cell wall within the path of protoplast cultures. Selected polysaccharides, such as hemicellulose (xyloglucan) and pectins (homogalacturonan, galactan, and arabinan), as well as proteins, were investigated due to their well-known involvement in various developmental processes, ranging from cell proliferation, differentiation, and expansion to somatic embryogenesis and plant growth [13, 16, 60, 61] (Fig. 4).

We noted a constitutive occurrence of hemicelluloses (galactosylated xyloglucan) and pectins (homogalacturonan methyl-esterified domains) in the walls of regenerating protoplasts and protoplast-derived cells. However, our results showed a changed distribution of RGI side chains, arabinan and galactan, in the analysed *Fagopyrum* species (Supplementary Fig. S5-S8). Many studies confirm that RGI side chain composition changes are related to cell differentiation status [20, 62]. The occurrence of galactan in the cell walls relates to their strengthening in some studied plant systems [63]. The appearance of the LM5 epitope on the 5th day of *Fagopyrum* cultures, just after cell division, could point to the strengthening of newly produced cell walls. Additionally, Potocka, et al. 2018 [20] noted the occurrence of galactan within the embryogenic domain of the *Arabidopsis* explant in cells presumably competent for somatic embryo formation. Therefore, we can suppose that the occurrence of galactans in inner cell walls on the 15th day of *F. tataricum* cultures can mark embryogenic domains within the cell colonies (Supplementary Fig. S5). Our results are also consistent with observations related to galactan-rich tobacco protoplasts that differentiated and were able to form new organs, compared to sugar beet protoplasts, which were poor in galactan and were not able to regeneration [16].

Arabinan side chains provide elasticity of cell walls, thus ensuring flexibility for intensively dividing cells [64], which could correlate with the presence of the LM6 epitope on the 5th day of *Fagopyrum* cultures (Supplementary Fig. S7-S8). Moreover, arabinan is important in regulating the water content during desiccation and in salt-tolerant species, preventing the irreversible aggregation of cell wall polymers [65]. Protoplast cultures constantly go through intensive development related to cell division, growth or elongation; therefore, the occurrence of arabinans ensures that elasticity is necessary. Additionally, the culture is susceptible to desiccation when the callus overgrows agarose beads at the end of the culture. It can explain the occurrence of the LM6 epitope on the 30th and 50th day of *F. esculentum* and *F. tataricum* cultures, respectively. So far, the occurrence of arabinans was noted in guard- and mesophyll-derived protoplasts of tobacco and only in protoplast-derived callus of sugar beet [15, 16]. In general, both galactan and arabinan are essential for proper protoplast regeneration.

Hydroxyproline-rich proteins such as highly glycosylated AGPs are involved in different developmental processes, from cell proliferation, differentiation and expansion to somatic embryogenesis and plant growth [66]. Because of the multifunctionality of AGPs, many studies regarding protoplast cultures detected AGPs during cell wall re-building and during protoplast development [13–16]. In our research, widespread occurrence of AGPs recognised by JIM13 antibody was detected in the cell wall and internal cell compartments (Table 1), which may indicate their role in many cellular processes. Our results are in accordance with reports noted for sugar beet, tobacco and carrot protoplast cultures where the abundant distribution of JIM13 antibody was noticed [14–16]. Interestingly, the abundant occurrence of AGPs in cytoplasmic compartments, e.g. vacuoles, can indicate carbohydrate turnover. Butowt, et al. 1999 [67] detected AGPs in the vacuoles of sugar beet protoplast cultures, which shows these components’ degradation. On the other hand, the localisation of AGPs detected by the JIM16 antibody differed between the analysed *Fagopyrum* species and the time points. In *F. tataricum* cultures, the JIM16 epitope was observed mainly in internal cell compartments until the 50th day, when the signal was undetected. For *F. esculentum* protoplast cultures, AGPs recognised by the JIM16 antibody were not localised at any point in the study. The noted differences are in accordance with the observed disappearance and enhanced expression of AGPs in some species undergoing cell differentiation [20, 68]. Godel-Jędrychowska, et al. 2019 [14] observed differences in AGPs expression in different carrot species pointing to cell-, tissue- and species specific AGPs presence.

Among AGP subclasses, fasciclin-like arabinogalactan proteins (FLAs) containing AGP-like glycated domains and fasciclin domains were detected by proteomic analysis. FLAs are proposed to play structural and signalling functions by organising cell wall components, regulating cell wall properties and affecting cell-to-cell interactions. Additionally, a relation between FLAs accumulation and cells’ embryogenic potential and involvement in competence acquisition during shoot organogenesis in tissue culture of *Arabidopsis* was reported [69]. In our research, the occurrence of FLAs between *F. esculentum* and *F. tataricum* varied (in *F. esculentum* cultures, an increase over time was observed for *F. tataricum* – a decrease; Fig. 3D). The higher FLAs accumulation on the 5th day of *F. tataricum* culture, as followed by a decrease in their accumulation, can point to the role of FLAs in controlling cell expansion and cell-cell interaction during cell colony formation. It suggests that FLAs modulate the organisation of cell wall polysaccharides during cell division events.

Extensins are moderately glycosylated proteins that participate in cell extension and provide a stabilising and reinforcing role in the wall of cells that have stopped elongating [70]. Lee, et al. 2013 [71] demonstrated both the presence of EXTs on the surface cells of embryogenic callus and protocorm-like bodies of *Phalaenopsis* and their absence in non-embryogenic callus cells, leading to reduced regeneration potential. Interestingly, protocorm-like bodies showed mainly epidermal localisation of these proteins. EXTs epitopes were also found in *Mussa* spp. AAA embryogenic cells, proembryos and globular embryos, especially in their cell walls and extracellular matrix [72]. Our results concerning EXTs presence on the surface of outer periclinal walls of the peripheral cells of *F. esculentum* protoplast-derived cultures (Supplementary Fig. S14) are in accordance with the examples mentioned above. EXTs presence was observed between the 10th and 20th day of culture in carrot protoplast cultures but disappeared at the end of the cultures [14]. However, in our research, we noted on the 5th day EXTs presence, which can explain EXTs participation in cell plate formation during cell division as it was reported for Arabidopsis *root-*,* shoot*,* hypocotyl-defective* (*rsh*) mutant [73, 74]. Like in carrot protoplast cultures, we also observed the disappearance of EXTs in *F. tataricum* cultures.

*CCOMT* is involved in reinforcing plant cell walls by participating in the lignin biosynthesis pathway, especially in forming cell wall ferulic esters [75]. In *F. esculentum* cultures, we noted a decrease in *CCOMT* expression among the analysed time points (Fig. 3D). Sharifi, et al. 2012 [76] showed a significant decrease in CCOMT accumulation in embryogenic callus compared to non-embryogenic callus of *Crocus sativus.* Authors suggest that higher expression of CCOMT in non-embryogenic callus may inhibit somatic embryogenesis by increasing cell wall lignification, thus strengthening the cell wall. In alfalfa plants, Guo, et al. 2001 [77] noted that down-regulation of *CCOMT* led to reduced lignin levels and accumulation of soluble caffeic acid β-D-glucoside. We can suspect that during the development of protoplast cultures, the cell wall is enriched in caffeic β-D-glucoside because CCOMT downregulation enables cell wall stretching.

## Materials and methods

### Protoplast isolation

Protoplasts were isolated from the EC of *F. esculentum* and the MC of *F. tataricum.* The callus lines were obtained from immature zygotic embryo for both species and maintained on RX medium according to Betekhtin, et al. 2017 [68] at 26 ± 1^o^C in the dark. The callus was subcultured every three weeks for *F. esculentum* and every two weeks for *F. tataricum*. The calli of both species exhibited different regeneration modes. EC of *F. esculentum* regenerated exclusively through somatic embryogenesis, whereas MC of *F. tataricum* regenerated via both organogenesis and somatic embryogenesis. All protoplast isolation and culture steps were performed according to the established protocol for *F. esculentum* [6] and *F. tataricum* [7].

At three time points of the culture: 5th, 10th and 30th or 50th day for *F. esculentum* and *F. tataricum*, respectively, the agarose beads were frozen in 5 ml Eppendorf’s and kept at -80^o^C to use as a material for proteomics and RT-qPCR. Fresh material was used for lipid staining. The material was fixed at the respective three-time points for immune- and histological analyses.

### Histological and immunostaining procedure

At each of the three time points representing characteristic cellular events, the beads of protoplast cultures were prepared according to the procedure described by Betekhtin, et al. 2019 [26] for histological and immunostaining study. The material was placed in a fixative mixture of 4% paraformaldehyde (PFA, POCH) and 1% glutaraldehyde (GA, POCH) in phosphate buffer saline (PBS, pH = 7.2), deaerated and incubated overnight at 4^o^C. Then the samples were washed three times with PBS (15 min each), dehydrated in increasing ethanol concentrations (10%, 30%, 50%, 70%, 90%, 100% *v/v*) two times for each concentration (30 min each) and gradually embedded in London Resin (LR White resin, Polysciences Inc.) according to Milewska-Hendel, et al. 2024 [78]. After that, the samples were cut into 1.5 μm thick sections using an EM UC6 ultramicrotome (Leica Biosystems) and put on glass slides coated with poly-L-lysine (Gerhard Menzel). For histology analysis, the slides were stained with 0.05% Toluidine Blue O (prepared based on water, Sigma-Aldrich) for 5 min, washed with distilled water and viewed in a brightfield microscope (AxioImager Z2 epifluorescence microscope, Zeiss).

Immunostaining started with applying blocking buffer (BB; 2% fetal calf serum and 2% bovine serum albumin in PBS) to the sections for 30 min at RT. Then, primary monoclonal antibodies (diluted 1:20 in BB; Plant Probes) were applied on the slides, and overnight incubation at 4^o^C was performed. The antibodies used for the current study are listed in the Supplementary Table S1. After that, the samples were washed three times in BB and incubated with the secondary antibody (AlexaFluor 488 goat anti-rat IgG, Jackson ImmunoResearch Laboratories; diluted 1:100 in the BB) for 1.5 h at RT. After that time, the slides were washed thrice in the BB and PBS, 5 min each. Next, fluorescent brightener 28 (FB28; 0.01%; diluted in PBS; Sigma-Aldrich) was applied for 5 min at RT to visualise cellulose in the cell wall. After that, the slides were washed thrice in the PBS and distilled ultrapure H2O for 5 min each. Slides were sealed by application of the Fluoromont mounting medium (Sigma-Aldrich) and stored at 4^o^C.

All of the photographs and observations were performed using an AxioImager Z2 epifluorescence microscope equipped with an AxioCam Mrm monochromatic camera (Zeiss) equipped with narrow-band filters for visualisation of AlexaFluor 488 and FB28 fluorescence.

### Lipid staining

The occurrence of lipid substances was detected by application of Sudan Black B and Sudan III (Sigma-Aldrich). Before staining, the material was fixed overnight at 4^o^C in a mixture of PFA and GA as described above and washed three times in PBS (5 min each) prior to lipid staining. For Sudan Black staining, the material was incubated in a 1% solution of Sudan Black B in 70% ethanol for 20 min in RT. In the case of Sudan III, the material was immersed in 0.5% staining solution for 2 h in RT. After staining, the material was washed in 50% ethanol and distilled water (three times, 5 min each). Observations were carried out with the use of a brightfield microscope (Zeiss).

### Total proteins isolation and LC-MS/MS analysis

The total proteins extraction was performed from material frozen at -80 °C. The isolation and further analysis were performed for four biological replications. The material was ground in liquid nitrogen with the addition of 100 mg/per sample of polyvinylpyrrolidone (PVP, average mol wt 40,000, Sigma-Aldrich). The proteins were isolated from grounded material according to the protocol by Wu, et al. 2014 [79] following the tissue disruption (step 1), trichloroacetic acid/acetone precipitation (steps 2–3) and sodium dodecyl sulfate extraction of proteins (steps 9–11). The protein extract was further purified and precipitated using the chloroform/methanol precipitation approach, as described by Wessel, et al. 1984 [80]. The proteins were resuspended in buffer (7 M urea, 2 M thiourea, 1.7% PMSF, 50 mM DTT). The proteins concentration was measured using Bradford Reagent (Sigma-Aldrich, B6916-500 M) with Albumin Standard (Thermo Scientific) for standard curve preparation. The analysis of protein samples using liquid chromatography tandem-mass spectrometry (LC-MS/MS) was performed according to Pinski, et al. 2021 [81]. Briefly, the samples were prepared using the FASP protocol, digested with trypsin and peptides were used for LC-MS/MS analysis. The obtained data were processed using the MaxQuantsoftware package (version 1.5.8.3) and Perseus platform. The protein sequences were downloaded from the Chinese National Genomics Data Center database (https://bigd.big.ac.cn/) for *F. esculentum* “Pintian4” (GWHBJBK00000000) and *F. tataricum* “Pinku1” (GWHBJBL00000000) references genomes [82]. Gene ontology analysis was performed by annotating the genes with eggNOG 5.0 [83] and GO Enrichment version 2.0.1 implemented in Galaxy Australia version 21.09 [84].

### RNA isolation and real-time qPCR

Total RNA was isolated from the material of *F. esculentum* and *F. tataricum* on the 5th, 15th, and 30th (for *F. esculentum*) and 5th, 15th, and 50th (for *F. tataricum*) day of protoplast-derived cultures. Total RNA was isolated using a FastPure Plant Total TNA Isolation Kit - Polysaccharides and polyphenolics-rich (Vazyme Biotech). RNA concentrations were measured using a Nano-Drop ND-1000 (NanoDrop Technologies). The DNA was removed from the RNA samples by digesting them with an RNase-free DNase Set (Qiagen). Maxima H Minus First Strand cDNA Synthesis Kit (Thermo Fisher Scientific) and oligo-dT primers were used to produce the cDNA. The cDNA was diluted four-fold with water and used in a qPCR reaction (2.0 µl). Analyses were performed using a LightCycler^®^ 480 SYBR Green I Master (Roche) according to reaction conditions described in Sala-Cholewa, et al. 2024 [85]. The primers were designed based on *F. esculentum* “Pintian4” and *F. tataricum* “Pinku1” references genomes with Primer3Plus (Supplementary Table S2, sheet 7). The control genes (SAND, ACTIN) had a constant expression level in all tissue samples. The Ct values were calculated using LinRegPCR software (version 11, Academic Medical Centre). The tissues for the Real-Time qPCR analysis were produced in three biological repetitions, and two technical replicates of each repetition were analysed. The relative expression level was calculated using 2^–∆∆CT^, where ∆∆C_T_ represents ∆C_T_^reference condition^ − ∆C_T_^compared condition^. The two-way ANOVA (*p* < 0.05) followed by Tukey’s honestly-significant-difference test (Tukey HSD-test) (*p* < 0.05) was used to calculate any significant differences between the experimental combinations. The graphs (Fig. 3) show the average values with the standard deviation (SD).

## Conclusions

This study provides a comprehensive analysis of the development of *Fagopyrum* protoplast-derived cultures, tracing the process from the initial cell division through to cell colony formation and the development of microcalli. Insights into the cell wall composition and expression profile of selected genes revealed changes that can correlate with regaining embryogenic competence during cell colony development. Proteomic analysis revealed an accumulation of storage and embryogenesis-related proteins. We demonstrated varied expressions of somatic embryogenesis-related genes and proteins. Based on this analysis, we distinguished seed storage proteins like VIC, OLEO, and SBP, which may play an important role in the somatic embryogenesis pathway of regeneration. The seed storage proteins seems to be connected with activation of TFs. Additionally, we confirmed changes in the cell wall composition during the development of cell colonies, indicating ongoing differentiation processes.

## Electronic supplementary material

Below is the link to the electronic supplementary material.

**Supplementary Material 1**: **Supplementary table S1**: Antibodies used for immunostaining of protoplast cultures and the epitopes they recognise in the cell wall and relevant references.

**Supplementary Material 2**: **Supplementary table S2**: The statistical analysis of proteomic data.

**Supplementary Material 3**: **Supplementary figure S1**: Immunolocalisation of LM25 epitope in *F. tataricum* protoplast cultures on the 5th (A-A”), 15th (B-B”), and 50th (C-C”) day of the culture. A’ red arrows point to the presence of epitope in cytoplasmic compartments; C’ and C” brown arrow points to a fluorescence signal detected on a surface of outer periclinal walls of the peripheral cells. *FB* fluorescent brightener. Scale bars 10 μm. **Supplementary figure S2**: Immunolocalisation of LM25 epitope in *F. esculentum* protoplast cultures on the 5th (A-A”), 15th (B-B”) and 30th (C-C”) day of the culture. *FB* fluorescent brightener. Scale bars 10 µm. **Supplementary figure S3**: Immunolocalisation of LM20 epitope in *F. tataricum* protoplast cultures on the 5th (A-A”), 15th (B-B”) and 50th (C-C”) day of the culture. C’ and C” brown arrows point to the presence of epitope on a surface of outer periclinal walls of the peripheral cells. *FB* fluorescent brightener. Scale bars 10 μm. **Supplementary figure S4**: Immunolocalisation of LM20 epitope in *F. esculentum* protoplast cultures on the 5th (A-A”), 15th (B-B”) and 30th (C-C”) day of the culture. B” yellow arrows point signal in the internal cell walls. *FB* fluorescent brightener. Scale bars 10 μm. **Supplementary figure S5**: Immunolocalisation of LM5 epitope in *F. tataricum* protoplast cultures on the 5th (A-A”), 15th (B-B”) and 50th (C-C”) day of the culture. B’ yellow arrows indicate a signal in the internal walls of the cell colonies. *FB* fluorescent brightener. Scale bars 10 µm. **Supplementary figure S6**: Immunolocalisation of LM5 epitope in *F. esculentum* protoplast cultures on the 5th (A-A”), 15th (B-B”) and 30th (C-C”) day of the culture. B’ blue arrows indicate the outer periclinal cell wall. *FB* fluorescent brightener. Scale bars 10 µm. **Supplementary figure S7**: Immunolocalisation of LM6 epitope in *F. tataricum* protoplast cultures on the 5th (A-A”), 15th (B-B”) and 50th (C-C”) day of the culture. B’ yellow arrows indicate the presence of the epitope in cell walls. *FB* fluorescent brightener. Scale bars 10 µm. **Supplementary figure S8**: Immunolocalisation of LM6 epitope in *F. esculentum* protoplast cultures on the 5th (A-A”), 15th (B-B”) and 30th (C-C”) day of the culture. C’ yellow arrows indicate the presence of the epitope in cell walls. *FB* fluorescent brightener. Scale bars 10 µm. **Supplementary figure S9**: Immunolocalisation of JIM13 AGPs epitope in *F. tataricum* protoplast cultures on the 5th (A-A”), 15th (B-B”) and 50th (C-C”) day of the culture. C’ purple arrows indicate nonspecific binding of the antibody to phenolics. *FB* fluorescent brightener. Scale bars 10 µm. **Supplementary figure S10**: Immunolocalisation of JIM13 AGPs epitope in *F. esculentum* protoplast cultures on the 5th (A-A”), 15th (B-B”) and 30th (C-C”) day of the culture. A’ and B’ white arrows indicate localisation of the epitope in the vacuole; C’ purple arrows indicate nonspecific binding of the antibody to phenolics; C’ yellow arrows point to fluorescence signal in the cell wall of peripheral cells. *FB* fluorescent brightener. Scale bars 10 µm. **Supplementary figure S11**: Immunolocalisation of JIM16 AGPs epitope in *F. tataricum* protoplast cultures on the 5th (A-A”), 15th (B-B”) and 50th (C-C”) day of the culture. B’ white arrow indicates localisation of the epitope in the vacuole; B’ red arrows points to the fluorescence signal in cytoplasmic compartments. *FB* fluorescent brightener. Scale bars 10 µm. **Supplementary figure S12**: Immunolocalisation of JIM16 AGPs epitope in *F. esculentum* protoplast cultures on the 5th (A-A”), 15th (B-B”) and 30th (C-C”) day of the culture. *FB* fluorescent brightener. Scale bars 10 µm. **Supplementary figure S13**: Immunolocalisation of JIM20 extensin epitope in *F. tataricum* protoplast cultures on the 5th (A-A”), 15th (B-B”) and 50th (C-C”) day of the culture. C’ red arrows indicate localisation of the epitope in the intercellular spaces; brown arrow point to the presence of epitope on the surface of the outer periclinal walls of the peripheral cells *FB* fluorescent brightener. Scale bars 10 µm. **Supplementary figure S14**: Immunolocalisation of JIM20 extensin epitope in *F. esculentum* protoplast cultures on the 5th (A-A”), 15th (B-B”) and 30th (C-C”) day of the culture. B’, C’ and C” brown arrows point to the presence of epitope on a surface of outer periclinal walls of the peripheral cells; B’ purple arrows indicate the phenolics; *FB* fluorescent brightener. Scale bars 10 μm. **Supplementary figure S15**: Lipid staining in *F. tataricum* protoplast cultures during three times points: 6 h after protoplast isolation (A, B); on the 5th (C, C’, D, D’) and 15th (E, F) day of the culture. Lipid droplets were stained black or blue after Sudan Black staining (A, C, C’, E); orange color is a positive reaction after Sudan III staining (B, D, D’, F). Red and black arrows indicate lipid droplets. Scale bars 10 μm. **Supplementary figure S16**: Lipid staining in *F. tataricum* protoplast cultures on the 50th day of the culture. Lipid droplets stained orange after Sudan III staining (A-C); black or blue after Sudan Black staining (D, D’, E). Red and black arrows indicate lipid droplets. Scale bars 20 μm (A, D, E); 10 μm (B, C, D’). **Supplementary figure S17**: Lipid staining in *F. esculentum* protoplast cultures during four times points: 6 h after protoplast isolation (A, B); on the 5th (C, D) and 15th (E, F) day of the culture. Lipid droplets stained black or blue after Sudan Black staining (A, C, E); orange after Sudan III staining (B, D, F). Red and black arrows indicate lipid droplets. Scale bars 10 μm. **Supplementary figure S18**: Lipid staining in *F. esculentum* protoplast cultures on the 30th day of the culture. Lipid droplets stained orange after Sudan III staining (A-C); black or blue after Sudan Black staining (D-F). Red and black arrows indicate lipid droplets. Scale bars 10 μm (B, C, E, F); 20 μm (A, D). **Supplementary figure S19**: The summary of proteomics analysis results. The Venn diagram shows protein presence in *F. tataricum* and *F. esculentum* on different days of protoplast cultures. The protein was designated to be present in the treatment if protein was detected in at least three out of four biological replications (A). The count of differentially accumulated proteins (DAPs) in *F. tataricum* and *F. esculentum* protoplast cultures at different days (B). The cluster maps of treatments for proteomics data for *F. tataricum* and *F. esculentum* (C).

## Data Availability

The mass spectrometry data were deposited at the ProteomeXchange Consortium via the MassIVE repository with the dataset identifier PXD055850.

## Abbreviations

AGPs: Arabinogalactan proteins
BB: Blocking buffer
BBM: BABY BOOM
BM: Basal medium
CCOMT: Caffeoyl-CoA O-methyltransferase
CLV3: CLAVATA
EC: Embryogenic callus
ENDO: Endochitynases
EP DC-8: Embryogenic protein DC-8-like
EXTs: Extensins
FLAs: Fasciclin-like arabinogalactan proteins
GA: Glutaraldehyde
LEA: Late embryogenesis abundant protein
LEC1: LEAFY COTYLEDON
MC: Morphogenic callus
OLEO: Oleosin
PBS: Phosphate buffer saline
PCCs: Phenolics-containing cells
PECCs: Proembryogenic cell complexes
PFA: Parafolmaldehyde
PSK: Phytosulfokine-α
RGI: Rhamnogalacturonan I
RT: Room temperature
SBP: Seed biotin-containing protein
SSPs: Seed storage proteins
SUS: SUCROSE SYNTHASE
TFs: Transcription factors
VIC: Vicilin
WUS: WUSCHEL
