# Supplementary material for: The cell colony development is connected with the accumulation of embryogenesis-related proteins and dynamic distribution of cell wall components in in vitro cultures of Fagopyrum tataricum and Fagopyrum esculentum
Source: BMC Plant Biol. 2025 Jan 24;25:102. doi: 10.1186/s12870-025-06119-3 (PMC11761224; doi:10.1186/s12870-025-06119-3)

Supplementary Figure S1. Immunolocalisation of LM25 epitope in *F. tataricum* protoplast cultures on the 5th (A-A"), 15th (B-B"), and 50th (C-C") day of the culture. **A'** red arrows point to the presence of epitope in cytoplasmic compartments; **C'** and **C"** brown arrow points to a fluorescence signal detected on a surface of outer periclinal walls of the peripheral cells. *FB* fluorescent brightener. Scale bars 10 µm

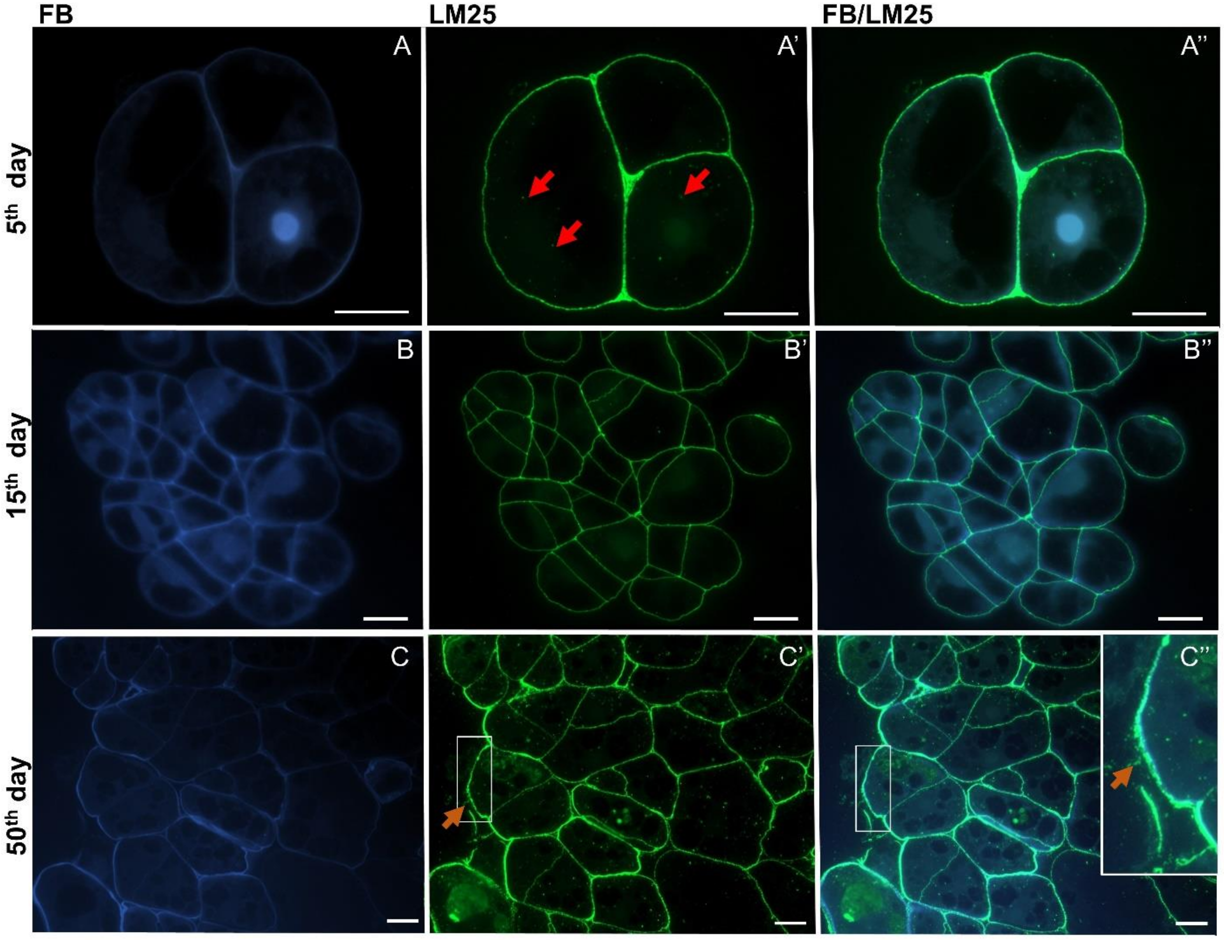

Supplementary Figure S2. Immunolocalisation of LM25 epitope in *F. esculentum* protoplast cultures on the 5th (A-A"), 15th (B-B") and 30th (C-C") day of the culture. *FB* fluorescent brightener. Scale bars 10 µm

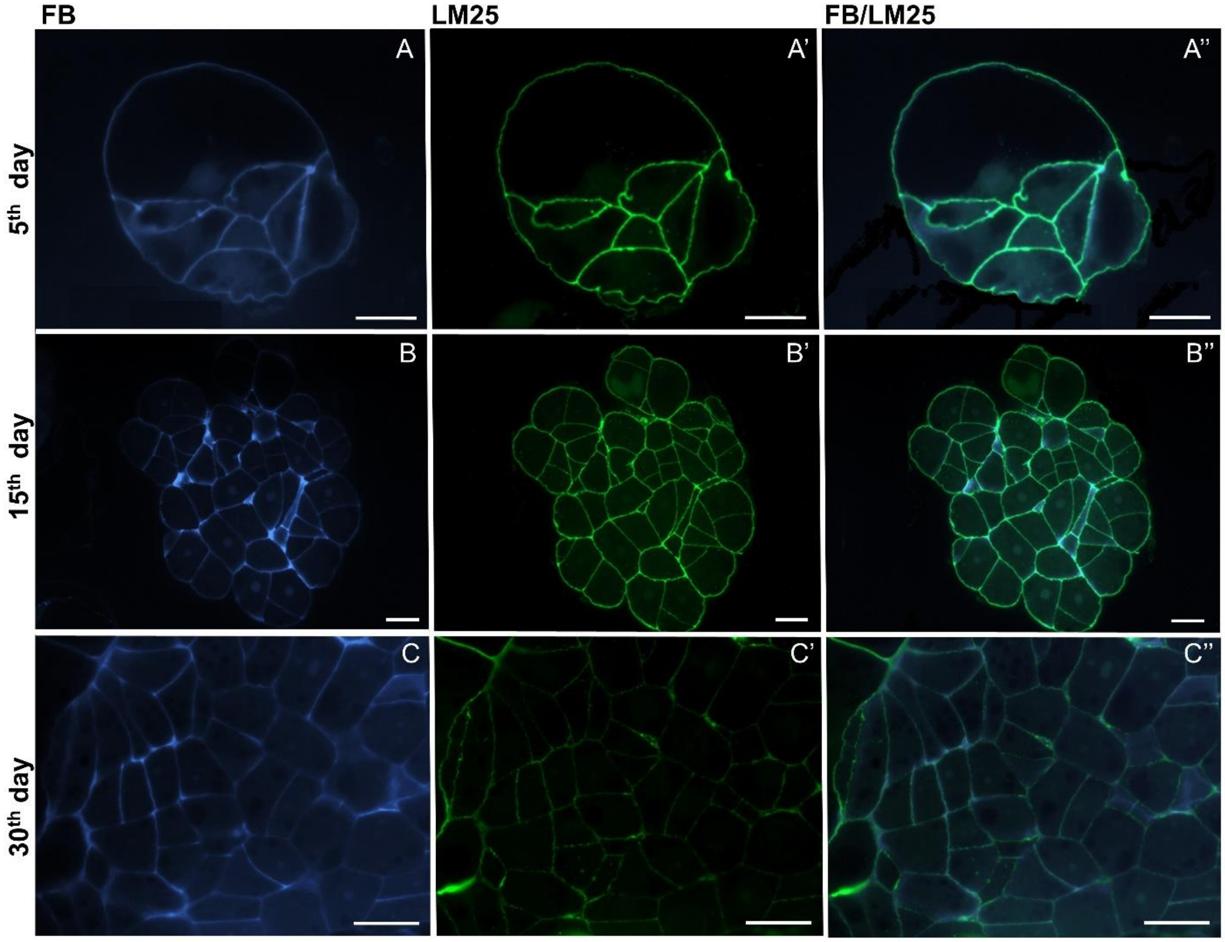

Supplementary Figure S3. Immunolocalisation of LM20 epitope in *F. tataricum* protoplast cultures on the 5th (A-A"), 15th (B-B") and 50th (C-C") day of the culture. **C'** and **C"** brown arrows point to the presence of epitope on a surface of outer periclinal walls of the peripheral cells. *FB* fluorescent brightener. Scale bars 10 µm

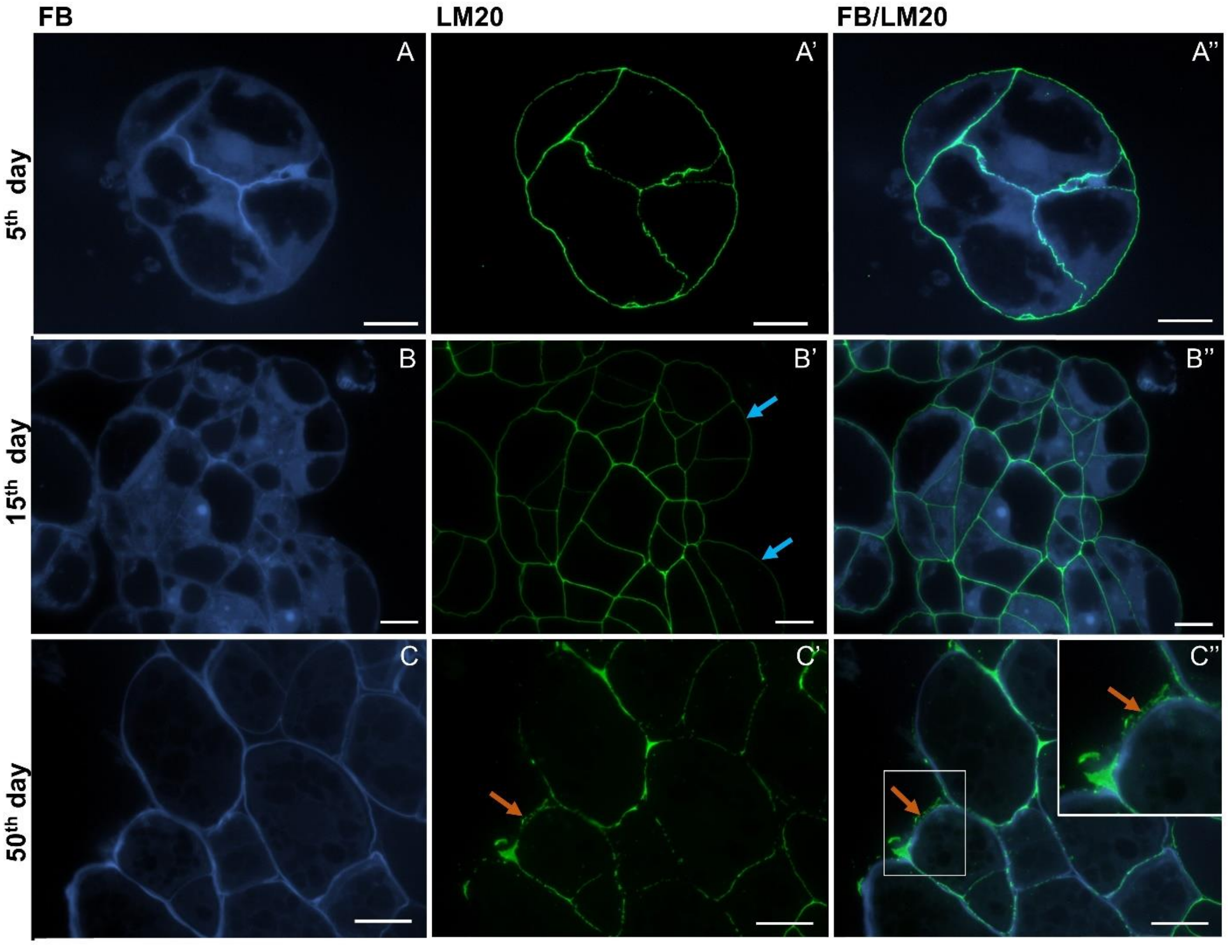

Supplementary Figure S4. Immunolocalisation of LM20 epitope in *F. esculentum* protoplast cultures on the 5th (A-A"), 15th (B-B") and 30th (C-C") day of the culture. **B"** yellow arrows point signal in the internal cell walls. *FB* fluorescent brightener. Scale bars 10 µm

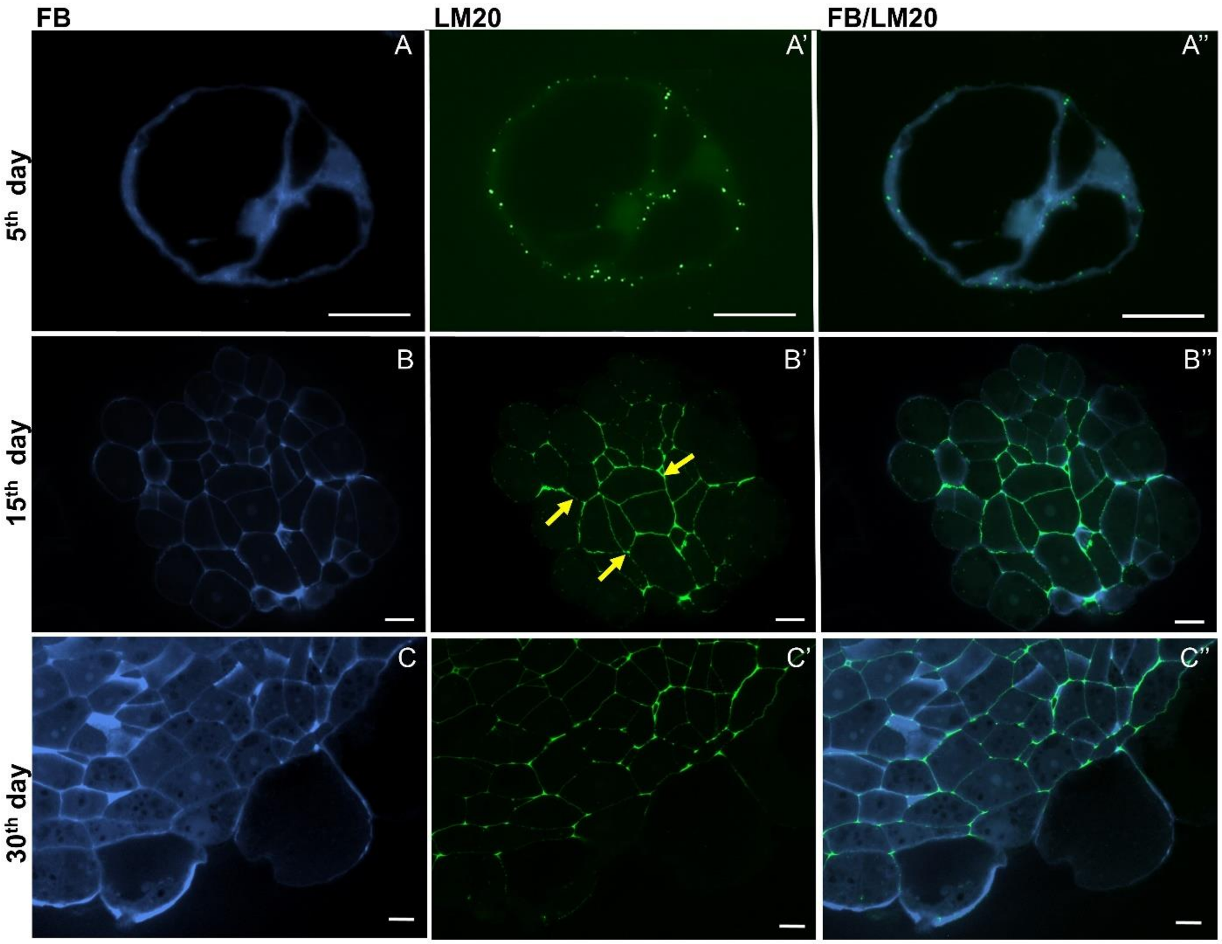

Supplementary Figure S5. Immunolocalisation of LM5 epitope in *F. tataricum* protoplast cultures on the 5th (A-A''), 15th (B-B'') and 50th (C-C'') day of the culture. **B'** yellow arrows indicate a signal in the internal walls of the aggregate. *FB* fluorescent brightener. Scale bars 10 µm

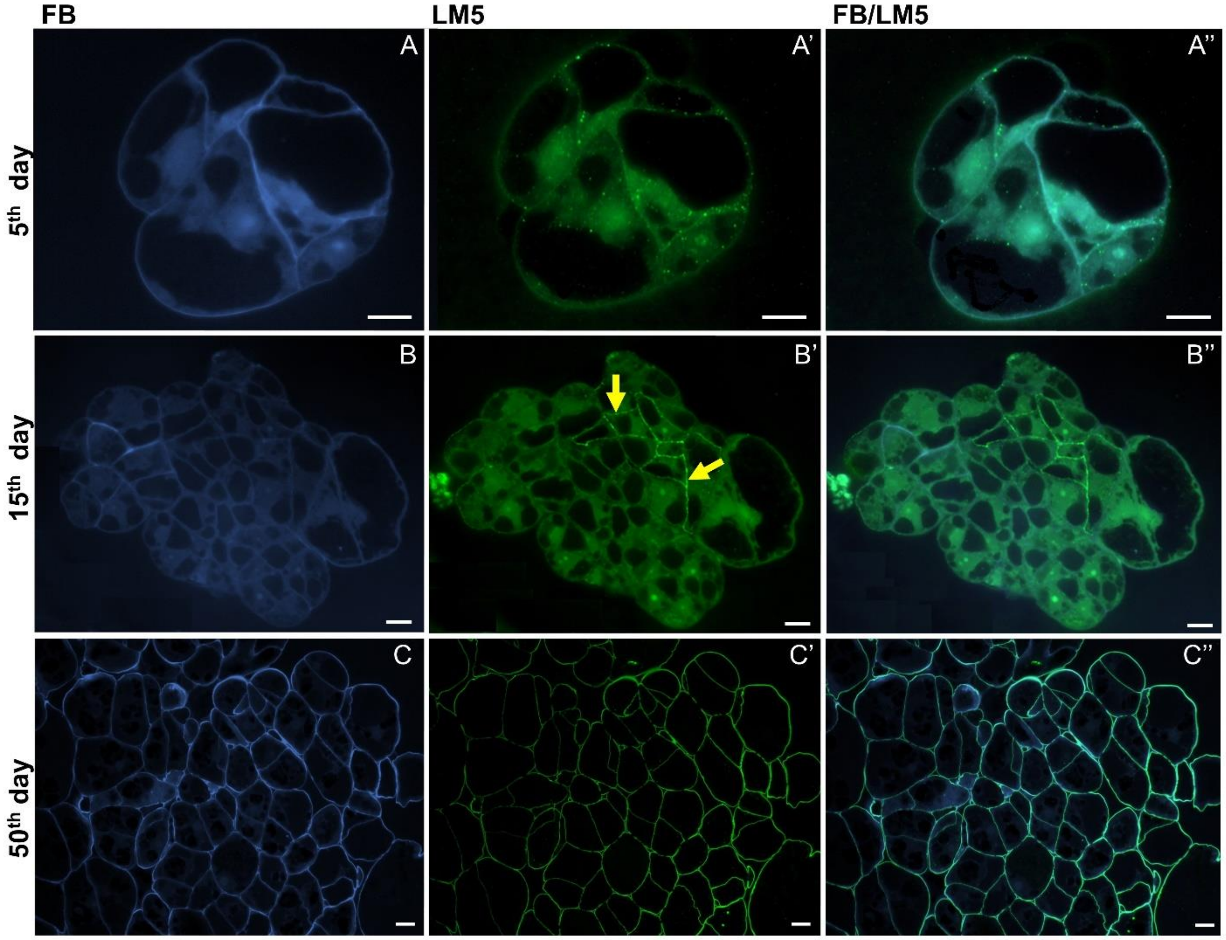

Supplementary Figure S6. Immunolocalisation of LM5 epitope in *F. esculentum* protoplast cultures on the 5th (A-A"), 15th (B-B") and 30th (C-C") day of the culture. **B'** blue arrows indicate the outer periclinal cell wall. *FB* fluorescent brightener. Scale bars 10 µm

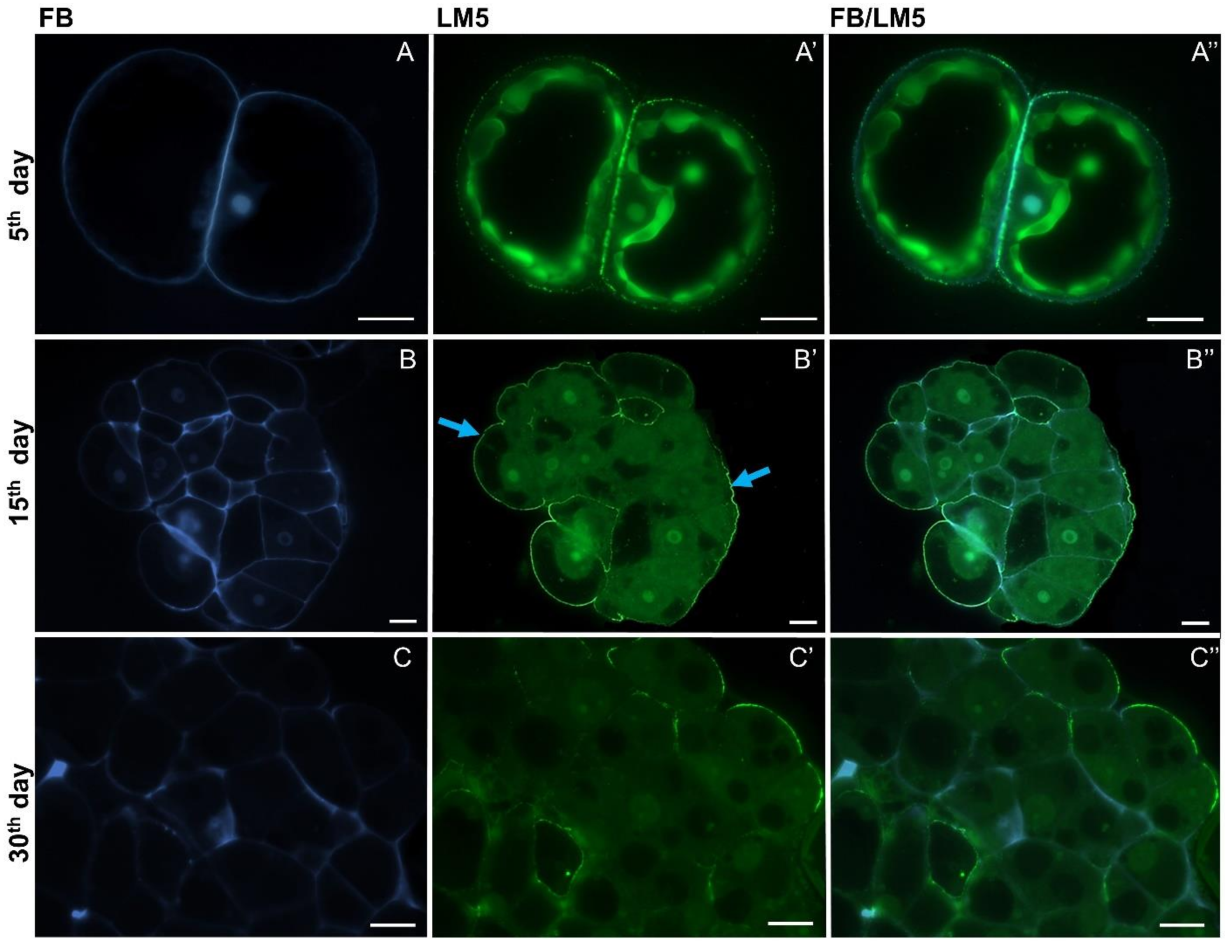

Supplementary Figure S7. Immunolocalisation of LM6 epitope in *F. tataricum* protoplast cultures on the 5th (A-A"), 15th (B-B") and 50th (C-C") day of the culture. **B'** yellow arrows indicate the presence of the epitope in cell walls. *FB* fluorescent brightener. Scale bars 10 µm

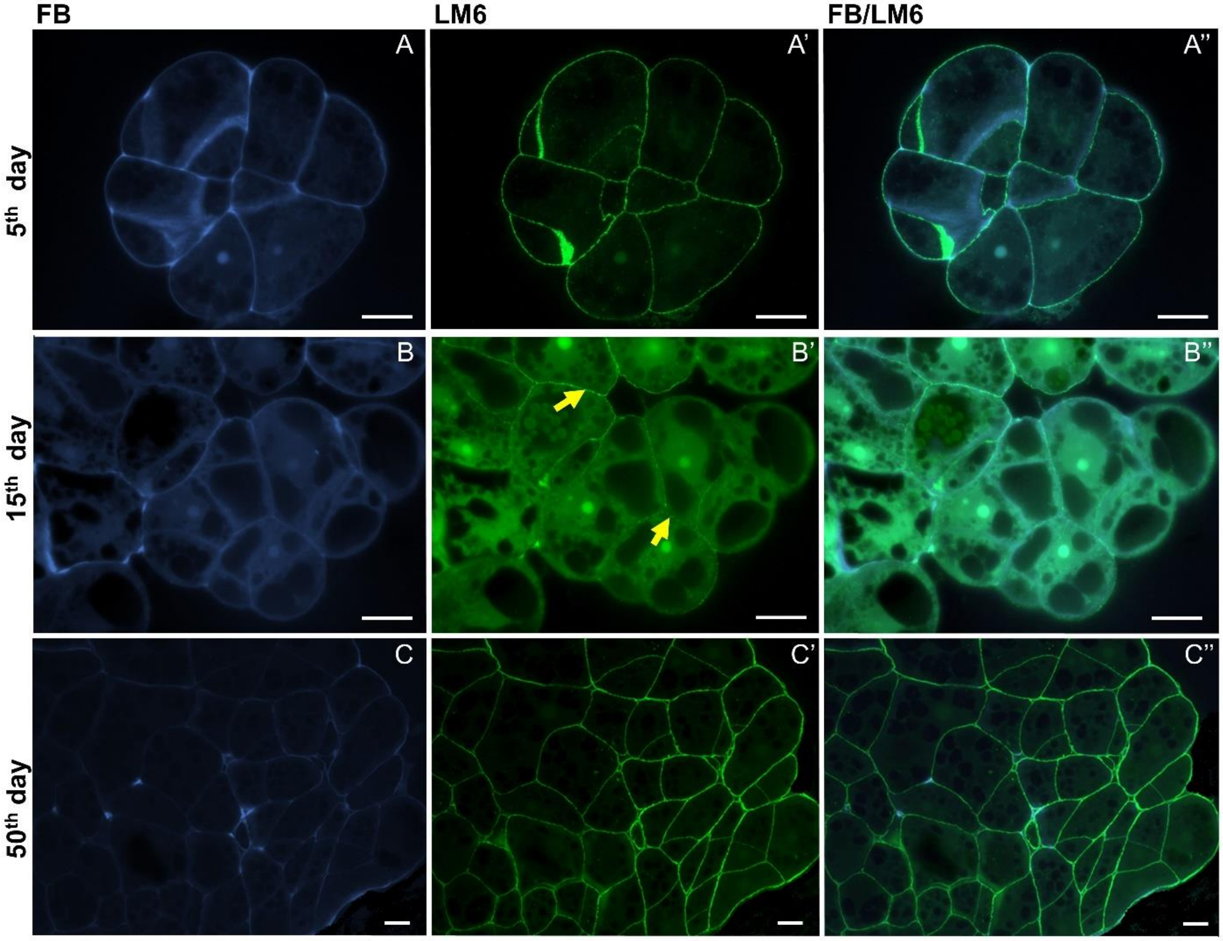

Supplementary Figure S8. Immunolocalisation of LM6 epitope in *F. esculentum* protoplast cultures on the 5th (A-A"), 15th (B-B") and 30th (C-C") day of the culture. **C'** yellow arrows indicate the presence of the epitope in cell walls. *FB* fluorescent brightener. Scale bars 10 µm

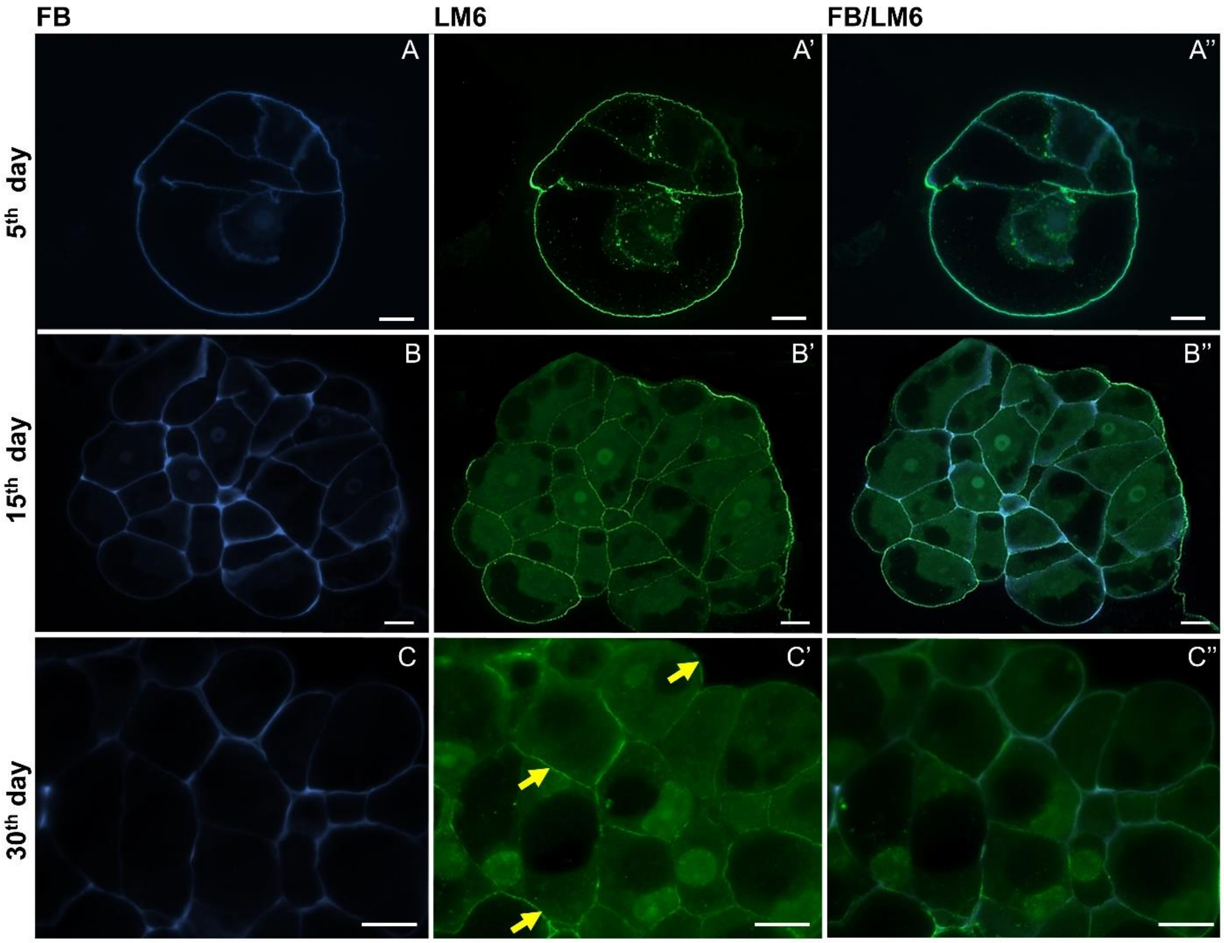

Supplementary Figure S9. Immunolocalisation of JIM13 AGPs epitope in *F. tataricum* protoplast cultures on the 5th (A-A''), 15th (B-B'') and 50th (C-C'') day of the culture. **C'** purple arrows indicate nonspecific binding of the antibody to phenolics. *FB* fluorescent brightener. Scale bars 10 µm

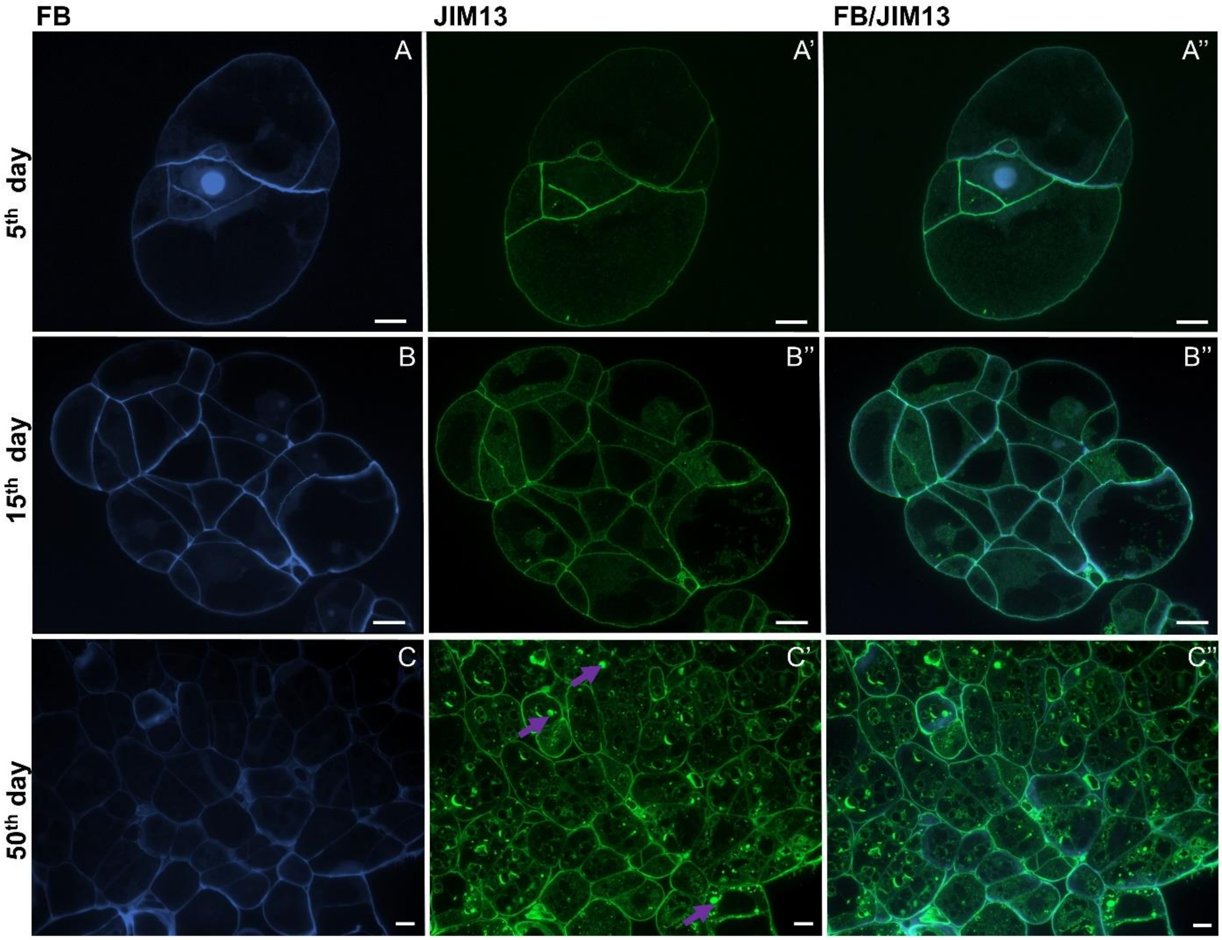

Supplementary Figure S10. Immunolocalisation of JIM13 AGPs epitope in *F. esculentum* protoplast cultures on the 5th (A-A"), 15th (B-B") and 30th (C-C") day of the culture. **A'** and **B'** white arrows indicate localisation of the epitope in the vacuole; **C'** purple arrows indicate nonspecific binding of the antibody to phenolics; **C'** yellow arrows point to fluorescence signal in the cell wall of peripheral cells. *FB* fluorescent brightener. Scale bars 10 µm

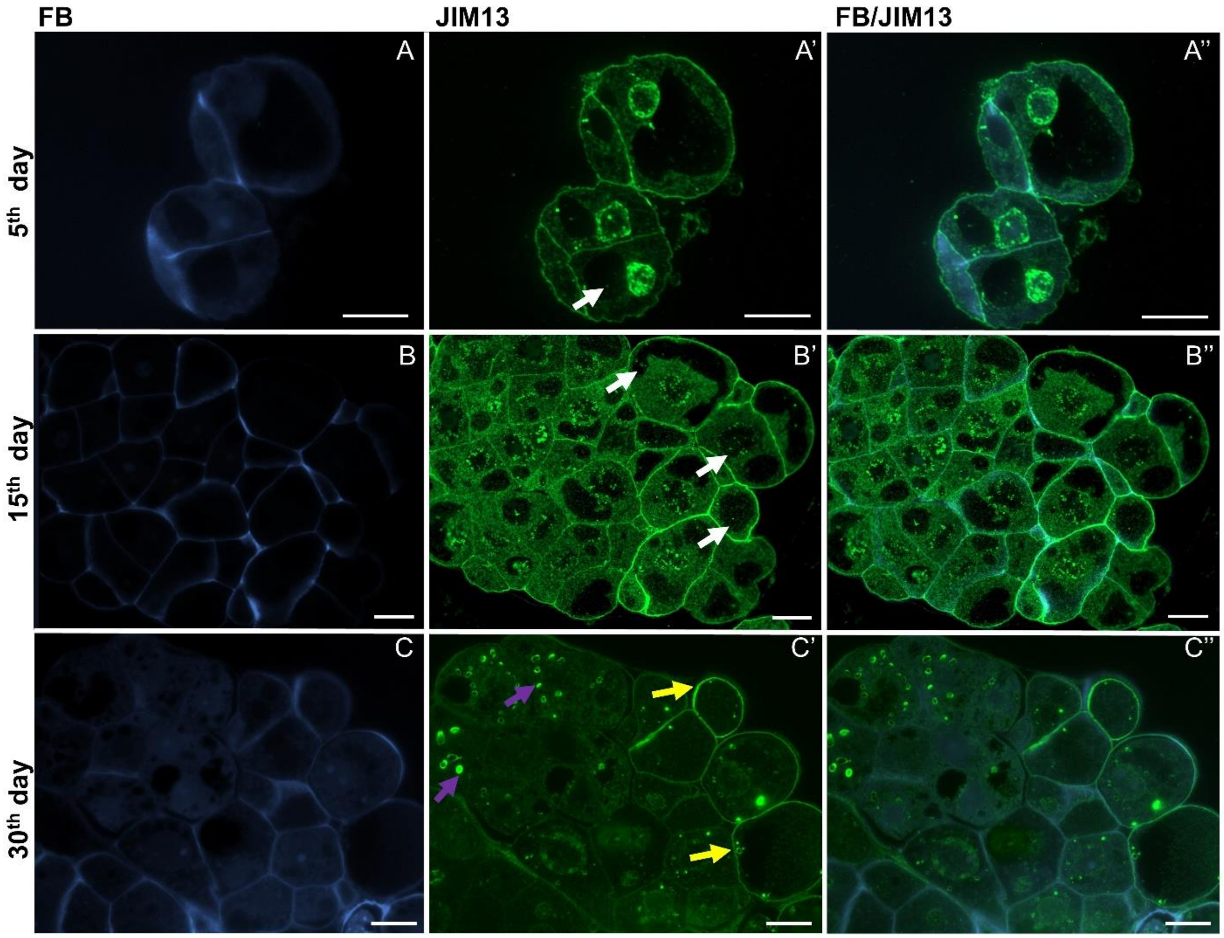

Supplementary Figure S11. Immunolocalisation of JIM16 AGPs epitope in *F. tataricum* protoplast cultures on the 5th (A-A"), 15th (B-B") and 50th (C-C") day of the culture. **B'** white arrow indicates localisation of the epitope in the vacuole; **B'** red arrows points to the fluorescence signal in cytoplasmic compartments. *FB* fluorescent brightener. Scale bars 10 µm

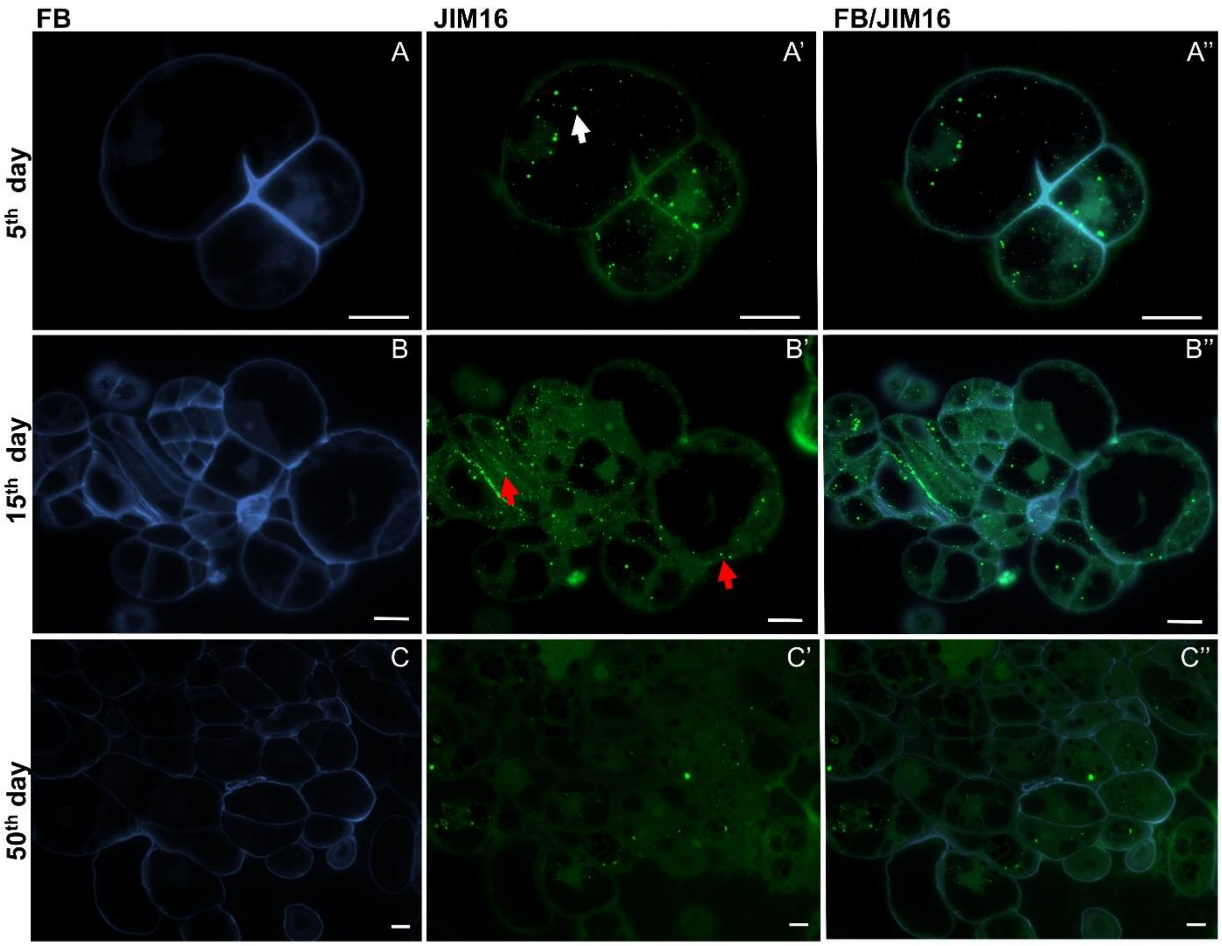

Supplementary Figure S12. Immunolocalisation of JIM16 AGPs epitope in *F. esculentum* protoplast cultures on the 5th (A-A"), 15th (B-B") and 30th (C-C") day of the culture. *FB* fluorescent brightener. Scale bars 10 µm

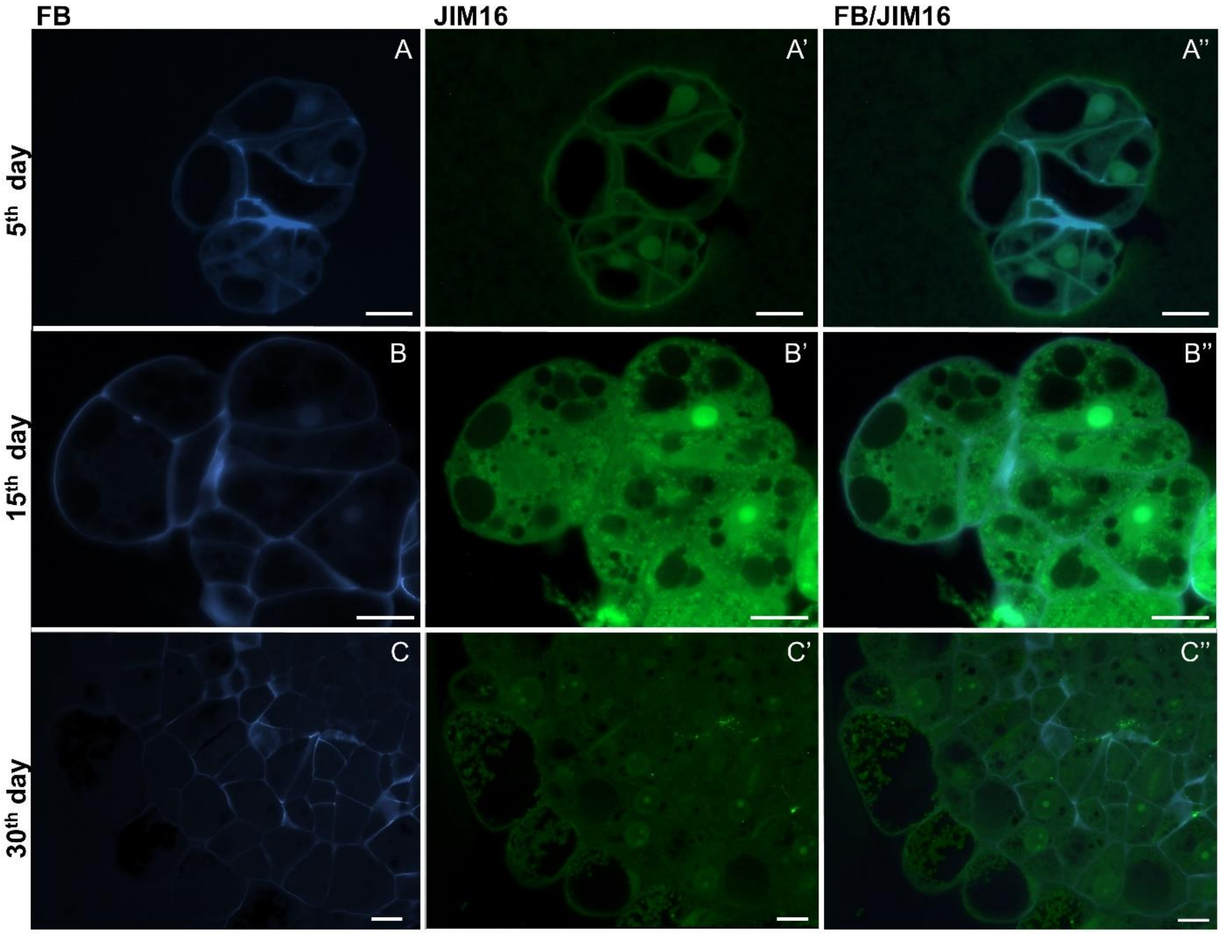

Supplementary Figure S13. Immunolocalisation of JIM20 extensin epitope in *F. tataricum* protoplast cultures on the 5th (A-A"), 15th (B-B") and 50th (C-C") day of the culture. **C'** red arrows indicate localisation of the epitope in the intercellular spaces; brown arrow point to the presence of epitope on the surface of the outer periclinal walls of the peripheral cells *FB* fluorescent brightener. Scale bars 10 µm

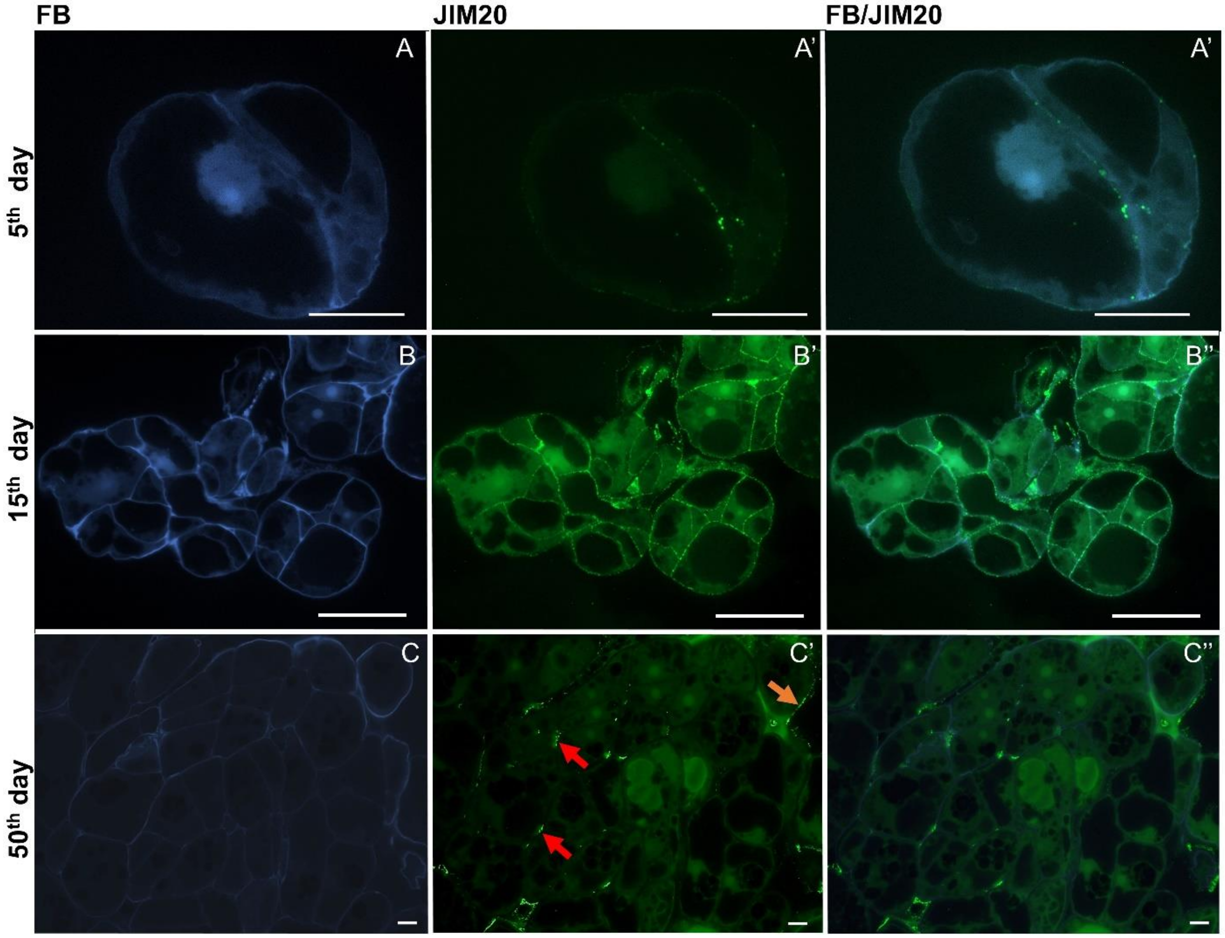

Supplementary Figure S14. Immunolocalisation of JIM20 extensin epitope in *F. esculentum* protoplast cultures on the 5th (A-A"), 15th (B-B") and 30th (C-C") day of the culture. **B', C'** and **C"** brown arrows point to the presence of epitope on a surface of outer periclinal walls of the peripheral cells; **B'** purple arrows indicate the phenolics; *FB* fluorescent brightener. Scale bars 10 µm

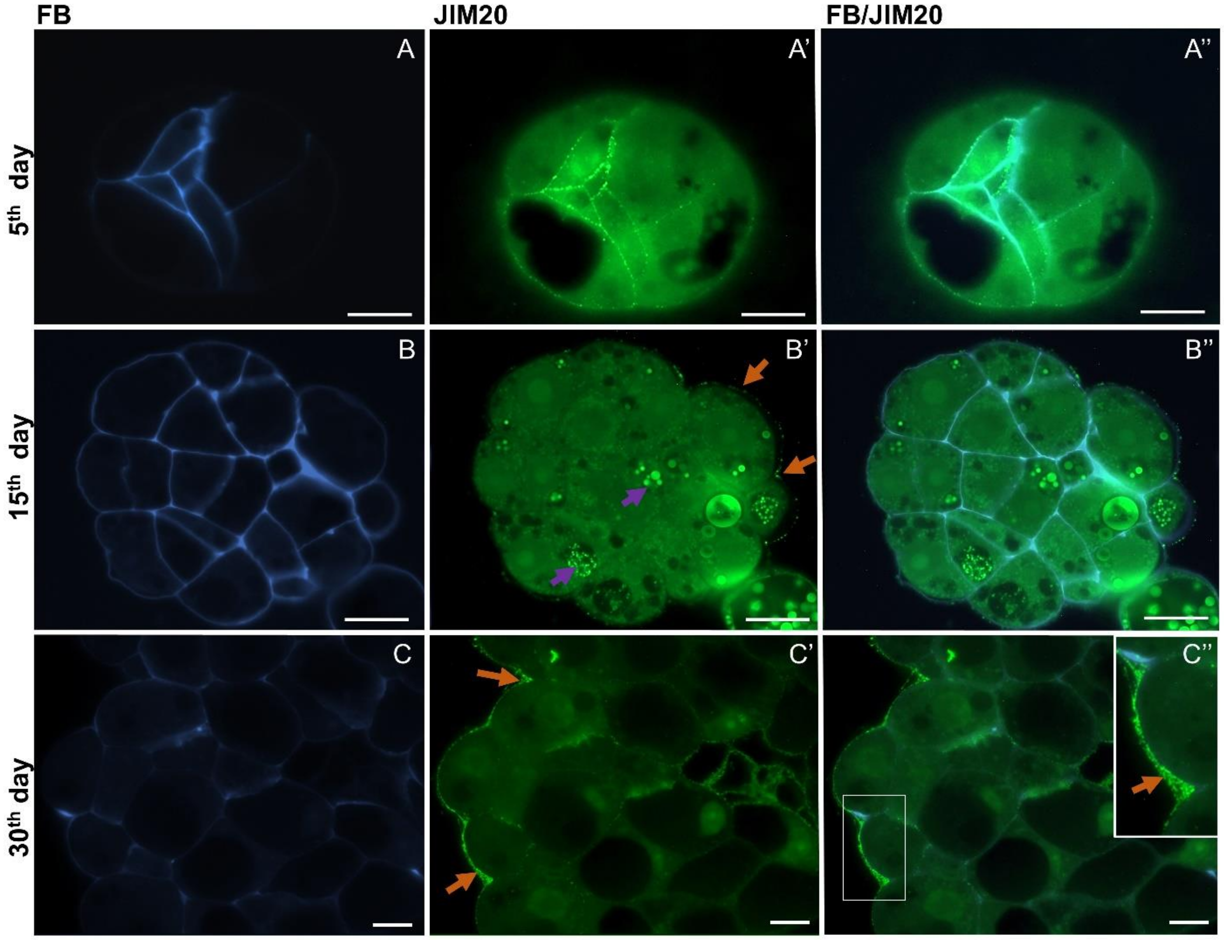

Supplementary Figure S15. Lipid staining in *F. tataricum* protoplast cultures during three times points: 6 hours after protoplast isolation (A, B); on the 5th (C, C', D, D') and 15th (E, F) day of the culture. Lipid droplets were stained black or blue after Sudan Black staining (A, C, C', E); orange color is a positive reaction after Sudan III staining (B, D, D', F). Red and black arrows indicate lipid droplets. Scale bars 10 µm

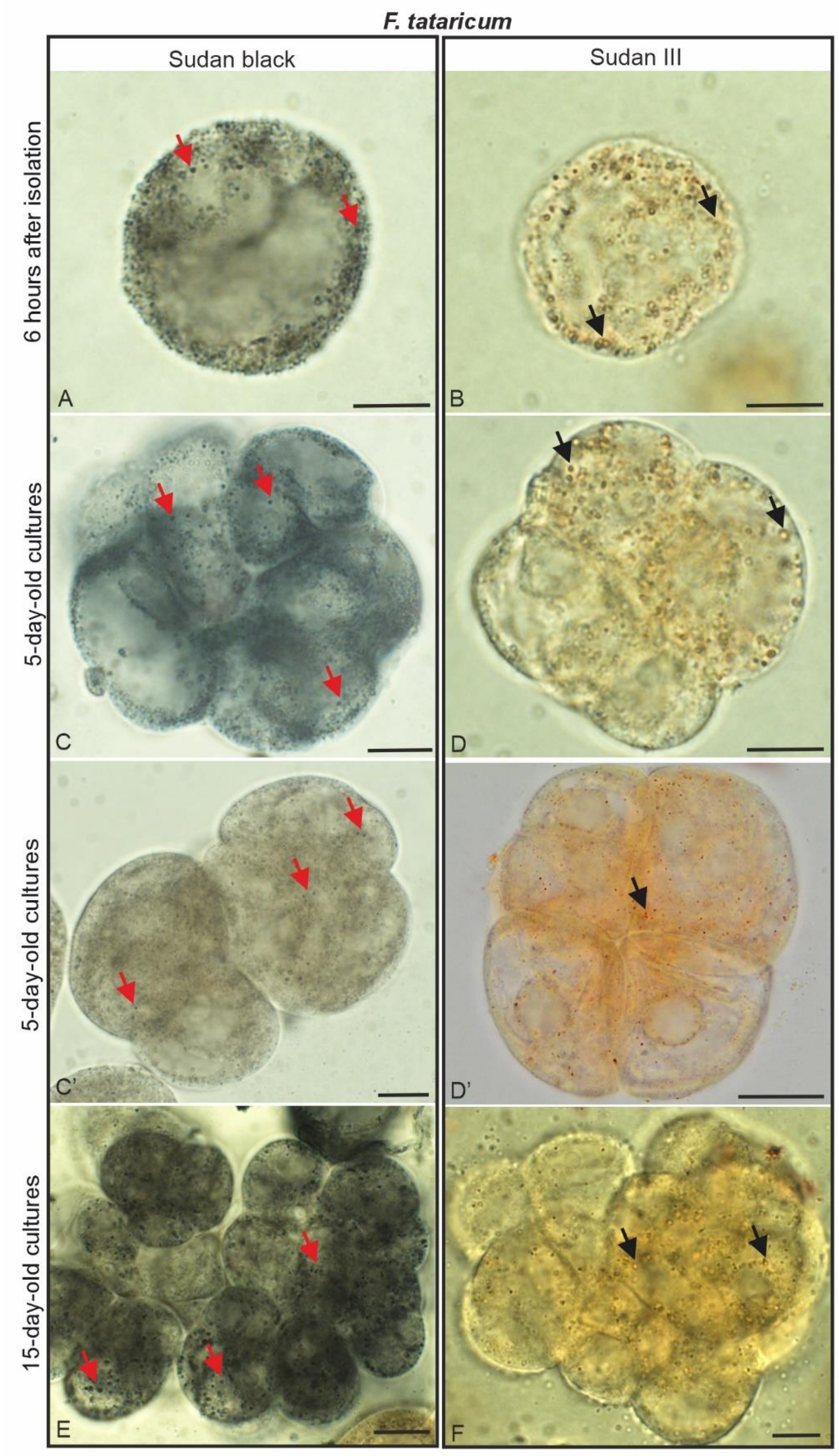

Supplementary Figure S16. Lipid staining in *F. tataricum* protoplast cultures on the 50th day of the culture. Lipid droplets stained orange after Sudan III staining (A-C); black or blue after Sudan Black staining (D, D', E). Red and black arrows indicate lipid droplets. Scale bars 20 µm (A, D, E); 10 µm (B, C, D')

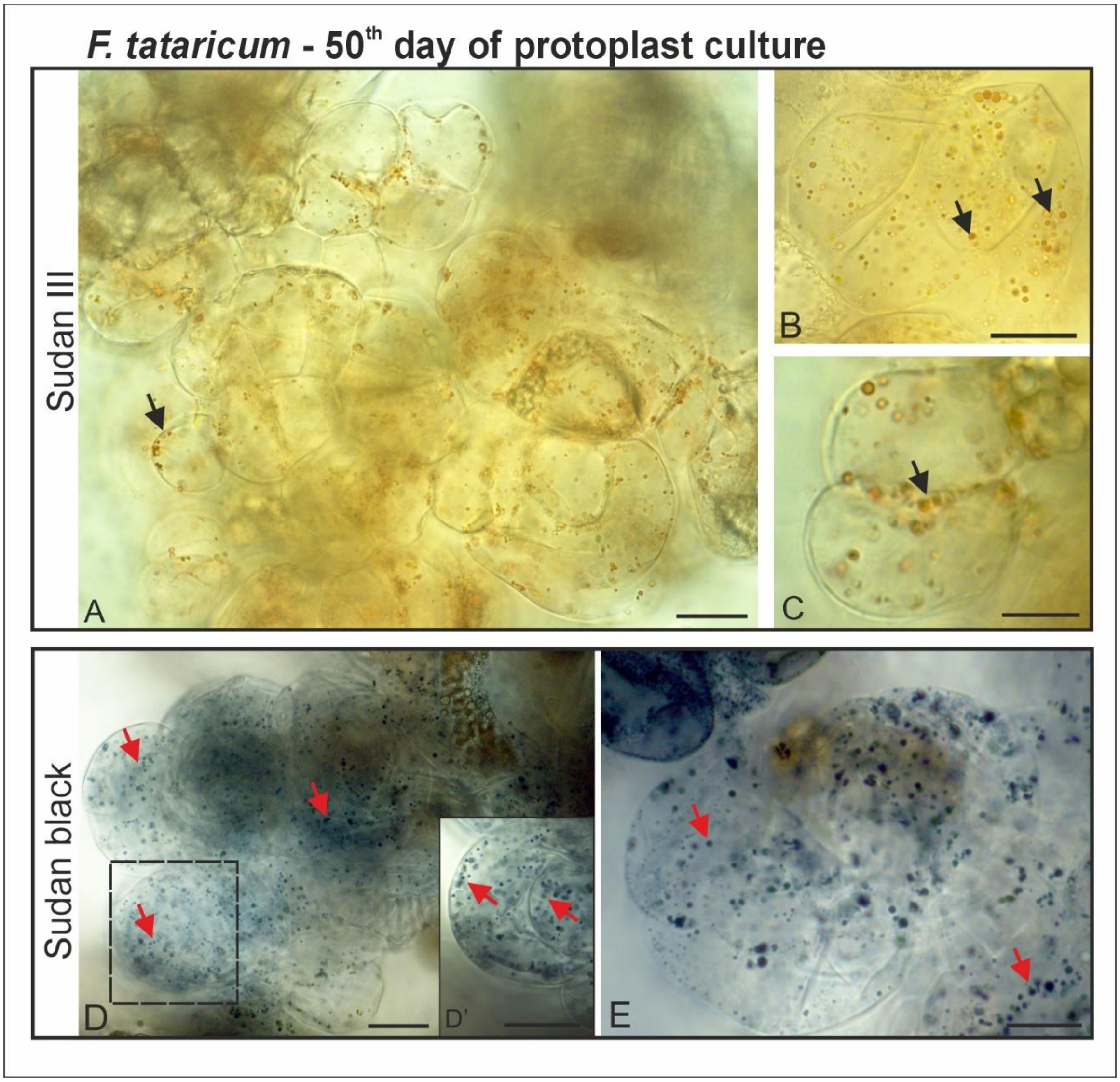

Supplementary Figure S17. Lipid staining in *F. esculentum* protoplast cultures during four times points: 6 hours after protoplast isolation (A, B); on the 5th (C, D) and 15th (E, F) day of the culture. Lipid droplets stained black or blue after Sudan Black staining (A, C, E); orange after Sudan III staining (B, D, F). Red and black arrows indicate lipid droplets. Scale bars 10 µm

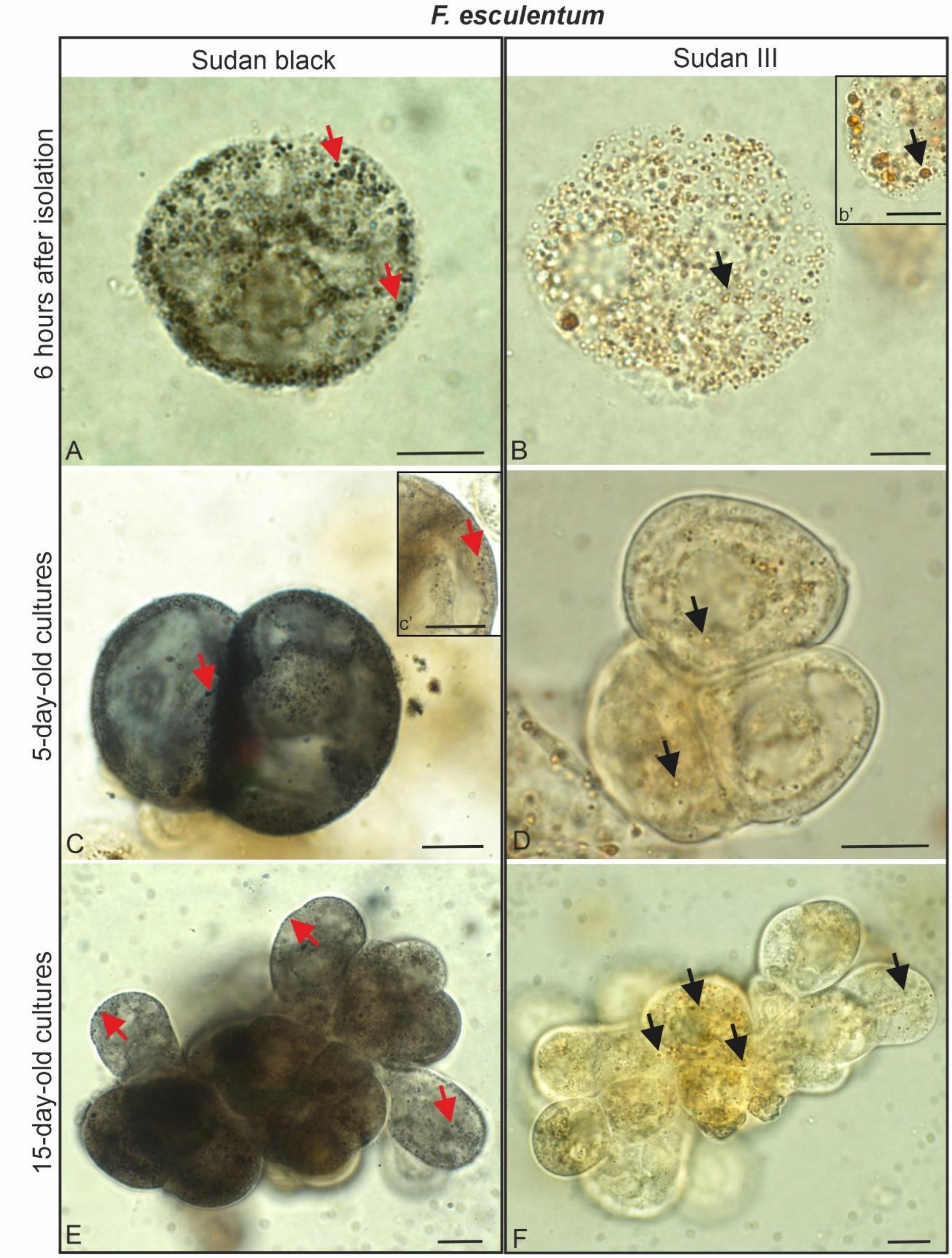

Supplementary Figure S18. Lipid staining in *F. esculentum* protoplast cultures on the 30th day of the culture. Lipid droplets stained orange after Sudan III staining (A-C); black or blue after Sudan Black staining (D-F). Red and black arrows indicate lipid droplets. Scale bars 10 µm (B, C, E, F); 20 µm (A, D)

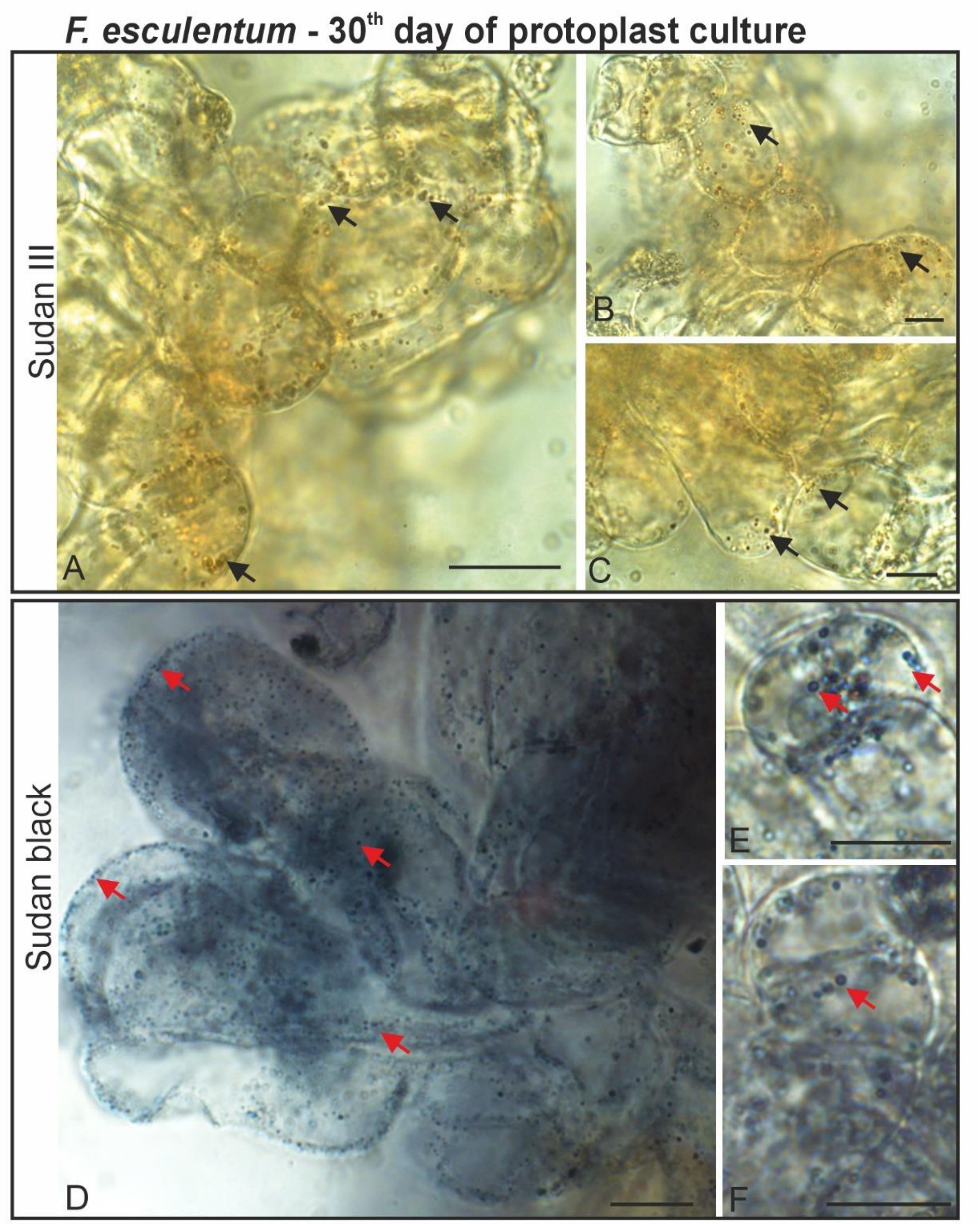

Supplementary Figure S19. The summary of proteomics analysis results. The Venn diagram shows protein presence in F. tataricum and F. esculentum on different days of protoplast cultures. The protein was designated to be present in the treatment if protein was detected in at least three out of four biological replications (A). The count of differentially accumulated proteins (DAPs) in *F. tataricum* and *F. esculentum* protoplast cultures at different days (B). The cluster maps of treatments for proteomics data for *F. tataricum* and *F. esculentum* (C)

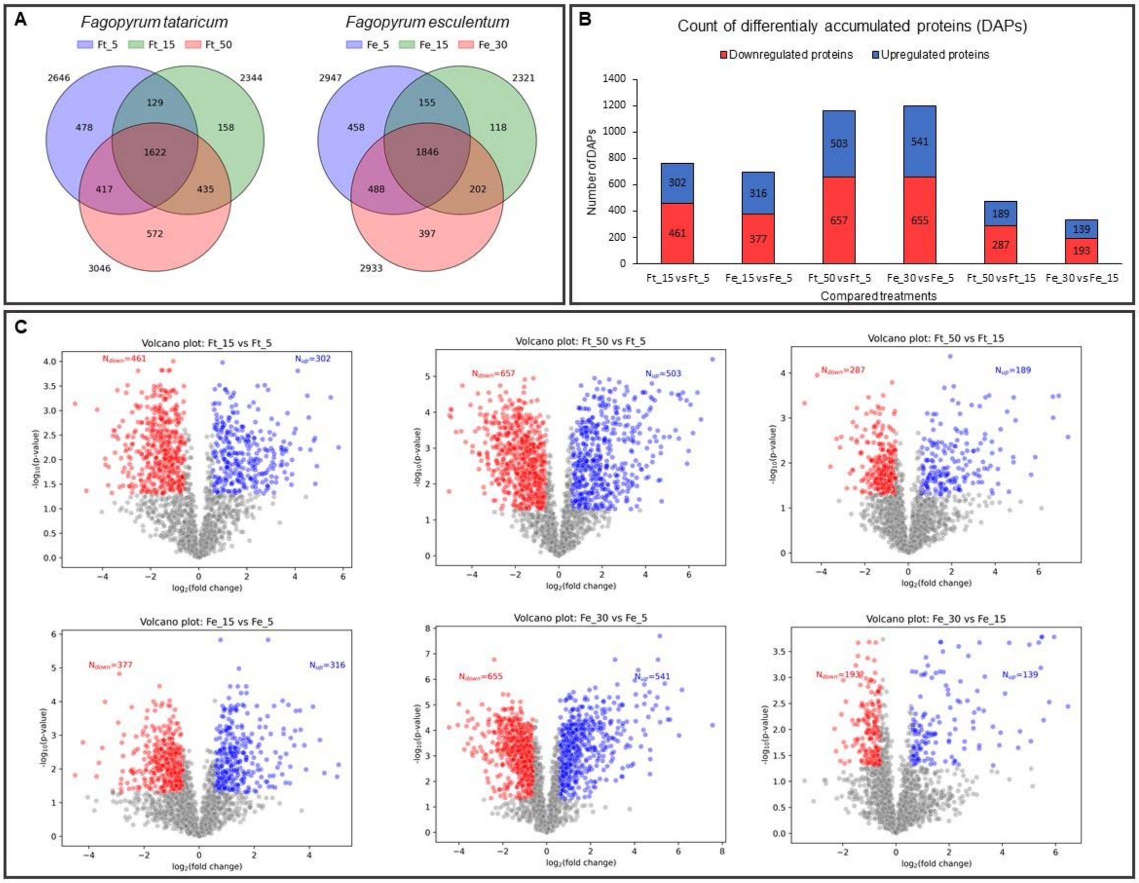

Supplement: Supplementary file 3 — Supplementary Material 3: Supplementary figure S1: Immunolocalisation of LM25 epitope in F. tataricum protoplast cultures on the 5th (A-A”), 15th (B-B”), and 50th (C-C”) day of the culture. A’ red arrows point to the presence of epitope in cytoplasmic compartments; C’ and C” brown arrow points to a fluorescence signal detected on a surface of outer periclinal walls of the peripheral cells. FB fluorescent brightener. Scale bars 10 μm. Supplementary figure S2: Immunolocalisation of LM25 epitope in F. esculentum protoplast cultures on the 5th (A-A”), 15th (B-B”) and 30th (C-C”) day of the culture. FB fluorescent brightener. Scale bars 10 µm. Supplementary figure S3: Immunolocalisation of LM20 epitope in F. tataricum protoplast cultures on the 5th (A-A”), 15th (B-B”) and 50th (C-C”) day of the culture. C’ and C” brown arrows point to the presence of epitope on a surface of outer periclinal walls of the peripheral cells. FB fluorescent brightener. Scale bars 10 μm. Supplementary figure S4: Immunolocalisation of LM20 epitope in F. esculentum protoplast cultures on the 5th (A-A”), 15th (B-B”) and 30th (C-C”) day of the culture. B” yellow arrows point signal in the internal cell walls. FB fluorescent brightener. Scale bars 10 μm. Supplementary figure S5: Immunolocalisation of LM5 epitope in F. tataricum protoplast cultures on the 5th (A-A”), 15th (B-B”) and 50th (C-C”) day of the culture. B’ yellow arrows indicate a signal in the internal walls of the cell colonies. FB fluorescent brightener. Scale bars 10 µm. Supplementary figure S6: Immunolocalisation of LM5 epitope in F. esculentum protoplast cultures on the 5th (A-A”), 15th (B-B”) and 30th (C-C”) day of the culture. B’ blue arrows indicate the outer periclinal cell wall. FB fluorescent brightener. Scale bars 10 µm. Supplementary figure S7: Immunolocalisation of LM6 epitope in F. tataricum protoplast cultures on the 5th (A-A”), 15th (B-B”) and 50th (C-C”) day of the culture. B’ yellow arrows indicate the presence of the [file 12870_2025_6119_MOESM3_ESM.pdf]
